# Household Gas Facility Detection Using RT-DETR with Morphology-Aware Feature Enhancement and Short-Axis-Aware Regression

**DOI:** 10.3390/s26216804

**Published:** 2026-10-28

**Authors:** Xufei Zhang, Dongxu Zhang

**Affiliations:** 1 Ulster College, Shaanxi University of Science and Technology, Xi’an 710021, China; 2 College of Mechanical and Electrical Engineering, Shaanxi University of Science and Technology, Xi’an 710021, China

**Keywords:** household gas safety, visual sensing, object detection, RT-DETR, dynamic snake convolution, local–global interaction, high-aspect-ratio object detection, bounding-box regression, small-object detection

## Abstract

Detecting household gas facility components in inspection images is challenging because of background clutter, partial occlusion, and substantial variation in object size and shape. This study presents an enhanced RT-DETR-R18 detector combining morphology-aware feature enhancement, local–global interaction, and geometry-sensitive regression. Gradient-Guided Morphology Enhancement (GGME) uses gradient-guided fusion of standard and directional dynamic-sampling branches to represent regular and elongated structures. A Bidirectional Local–Global Interaction Encoder (BLGIE) adaptively combines local structural cues with global context. Short-Axis-Aware Projection Regression (SAPR) supplements the original regression losses with weighted one-dimensional projection alignment and shape regularization. Experiments use 21,813 images containing 47,169 annotated instances across eight categories, with scene-level separation between training and validation sets. Relative to RT-DETR-R18, the proposed detector increases COCO-style mean average precision (mAP) from 67.67% to 71.08%, AP at an intersection-over-union threshold of 0.75 (AP75) from 74.49% to 79.87%, and small-object AP from 40.60% to 49.28%. With 22.3 million parameters and 63.7 GFLOPs, it achieves 72.3 frames per second on an NVIDIA GeForce RTX 4060 Laptop GPU at 640 × 640 resolution using batch-1 FP32 inference. These results indicate improved detection on the evaluated validation set, particularly for stricter localization and small objects.

## 1. Introduction

Household gas safety is an important component of urban public safety and residential risk management. Gas equipment and its combustion, exhaust, connection, fixation, protection, and control components are widely distributed in indoor residential environments. Non-standard installation, component aging, loose connections, and abnormal operating conditions may create safety risks that cannot be sufficiently identified by remote monitoring alone. Regular in-home inspection is therefore essential for discovering visible hazards and ensuring safe residential gas use. As the number of residential gas users increases and safety-management procedures become more standardized, conventional inspection workflows face increasing pressure in terms of efficiency, consistency, and traceability. Camera-based visual sensing provides a non-contact and scalable means of recording inspection evidence, localizing key facilities, and supporting computer-assisted recognition [1,2,3,4], and can serve as an important visual front end for portable and mobile inspection systems.

Traditional household gas inspection is primarily performed manually. Inspectors enter residential environments, locate gas-related facilities, examine their visible installation status, record inspection information, and determine whether further professional testing is required. Although manual inspection remains indispensable in practical applications, it is labor-intensive, and its results can be affected by inspector experience, attention, and operating habits. Differences in image acquisition, inspection order, and subjective judgment may also reduce the consistency of large-scale inspection records. Conventional image-processing methods based on color thresholds, edges, contours, handcrafted texture descriptors, or geometric rules can provide assistance under controlled conditions. In real residential scenes, however, illumination variation, metallic reflection, stains, blur, shadows, cluttered backgrounds, viewpoint changes, and overlapping facilities substantially reduce the reliability of handcrafted features and fixed decision rules. Stable component localization in uncontrolled household environments therefore remains a challenging visual-sensing problem.

The household gas facility detection task studied in this work includes eight target categories: Water_heater, Flue, Stove, In_needle, Ft_protection, Rubber_hose, Pipe_clamp, and Valve. These categories differ markedly in appearance, scale, geometric morphology, and dependence on contextual information. Water heaters and stoves are typically large and relatively regular in shape, whereas inlet needles, protection devices, pipe clamps, and valves occupy smaller image regions and may be partially occluded by surrounding pipes, device housings, connectors, or other components. Rubber hoses exhibit more distinctive geometric properties: they are flexible, slender, frequently curved, and highly variable in orientation. Their local appearance can also be confused with pipes, cables, device edges, and background contours. Intersections among hoses, valves, pipe clamps, and rigid pipes further weaken object boundaries. A detector for this task must therefore preserve conventional texture representation for regular objects while adaptively adjusting feature sampling, contextual interaction, and bounding-box localization for slender, curved, and partially visible structures.

Recent advances in deep learning have substantially promoted automated visual inspection. Convolutional neural networks can learn hierarchical semantic features directly from red-green-blue (RGB) images and have been applied to pipeline inspection, defect recognition, equipment damage analysis, and other industrial visual-sensing tasks [5,6,7,8,9,10,11,12,13]. More task-specific studies have explored You Only Look Once (YOLO)-based gas-facility recognition, camera-based valve-status monitoring, and image/ultrasonic pipe-leak localization in gas-related or process-industry environments [3,4,10,11,14]. Single-stage detectors provide high inference efficiency and are widely used in real-time applications, while multi-scale feature representation supports the detection of objects with different sizes [15,16,17,18]. Nevertheless, existing gas-related computer-vision studies mainly focus on pipeline defects, corrosion, cracks, leakage-related phenomena, valve-state monitoring, or isolated industrial components. Household gas facility detection differs from these tasks because it requires simultaneous localization of multiple facility components with heterogeneous appearances and morphologies in uncontrolled indoor scenes, rather than the recognition of a single defect type or the monitoring of a single equipment state on relatively uniform surfaces.

Transformer-based detectors provide another important route for object detection [19]. Detection Transformer (DETR) formulates detection as an end-to-end set prediction problem and removes manually designed anchors and non-maximum suppression (NMS) through bipartite matching [20]. Deformable DETR improves convergence and multi-scale feature modeling by applying sparse attention around reference points [21]. DETR with Improved DeNoising Anchor Boxes (DINO) further enhances query initialization and de-noising training, improving the stability of end-to-end detector training [22]. Real-Time Detection Transformer (RT-DETR) proposes an efficient hybrid encoder that decouples intra-scale feature interaction from cross-scale feature fusion and uses uncertainty-minimal query selection to generate high-quality initial queries for the Transformer decoder [23]. RT-DETRv2 and RT-DETRv3 subsequently improve training flexibility, deployment practicality, and dense positive supervision [24,25]. The clear backbone, hybrid encoder, query selection, and decoder stages in RT-DETR provide suitable intervention points for task-oriented improvements without redesigning the entire end-to-end detection pipeline [23,26,27,28].

Morphology-adaptive feature sampling is particularly relevant to the detection of elongated and deformable structures. Standard convolution samples features on a fixed rectangular grid, which is efficient but may introduce background responses when the target is narrow, curved, or partially separated from surrounding structures. Deformable convolution improves spatial adaptability by learning sampling offsets [29], and its modulated version further expands the scope of deformation modeling [30]. Dynamic Snake Convolution (DSConv) introduces directionally continuous sampling trajectories for tubular or curvilinear structures [31]. However, most existing dynamic or deformable sampling methods are designed for generic feature adaptation or pixel-level tubular-structure segmentation, whereas household gas facility detection requires simultaneous recognition of regular equipment, compact components, and flexible elongated objects. Directly replacing standard convolution with morphology-specific operators may therefore weaken the representation of large regular categories. Motivated by this observation, Gradient-Guided Morphology Enhancement (GGME) retains a standard convolution branch and introduces horizontal and vertical dynamic snake branches as complementary morphology-aware feature samplers, with feature-gradient cues used to guide spatial branch selection.

Local–global interaction has been widely studied in visual recognition through non-local operations [32], relation modeling [33], window-based hierarchical attention [34], focal attention mechanisms [35], and channel/spatial attention modules [36,37]. These methods enhance long-range dependency modeling and improve contextual representation. In partially occluded household gas scenes, however, local and global features may have different reliability: local features preserve visible fragments such as edges, connectors, and boundaries, whereas global features provide semantic context but may also introduce misleading associations from nearby pipes, walls, or appliances. Simple feature addition or concatenation does not explicitly model this reliability difference. Bidirectional Local–Global Interaction Encoder (BLGIE) is therefore designed as an asymmetric local–global interaction module. It uses global semantics to recalibrate local channels, local structural evidence to recalibrate global spatial responses, and discrepancy-guided fusion to combine the calibrated representations. The learned gate is not treated as an explicit occlusion probability map; rather, it estimates the relative reliability of local and global representations under detection supervision.

Bounding-box regression losses have also been extensively studied. Generalized intersection over union (GIoU) extends intersection over union (IoU) by introducing an enclosing-region penalty for non-overlapping boxes [38], while distance intersection over union (DIoU) and complete intersection over union (CIoU) incorporate center-distance and aspect-ratio constraints [39]. Other variants further introduce direction-aware or difficulty-adaptive penalties to improve localization stability [40,41]. These losses improve general bounding-box regression, but most of them still evaluate two-dimensional overlap or global geometric consistency without explicitly emphasizing the axis that is most sensitive for elongated horizontal boxes. For high-aspect-ratio objects, such as rubber hoses, a small displacement along the short axis can cause a disproportionate decrease in overlap even when the long-axis extent is accurately estimated. Short-Axis-Aware Projection Regression (SAPR) addresses this issue by decomposing localization into one-dimensional projected alignment along the x- and y-axes, assigning a larger weight to the ground-truth short-axis projection, and adding an elongation-gated shape-consistency term. In this way, SAPR complements rather than replaces the original L1 and GIoU objectives.

Despite this progress, existing general-purpose detectors still struggle to simultaneously handle regular equipment, slender flexible structures, partial occlusion, and geometry-sensitive localization in household gas scenes. Fixed-grid standard convolution may introduce background responses when processing narrow and curved rubber hoses, whereas relying only on morphology-specific operators may weaken conventional texture representation for regular objects. Partial occlusion removes discriminative local evidence, and simple addition or concatenation of local and global features does not explicitly estimate their relative reliability. In addition, existing IoU-based losses consider two-dimensional overlap, center distance, and side-length differences, but do not explicitly strengthen localization along the short axis of high-aspect-ratio targets. These observations motivate coordinated improvements in direction-aware morphology-adaptive feature extraction, selective local–global feature fusion, and short-axis-aware bounding-box regression.

In summary, household gas facility detection involves three interrelated challenges. The first is mixed-morphology representation: the detector must adapt to flexible, slender, and curved rubber hoses without degrading the representation of large regular equipment and compact components. The second is contextual reasoning under partial occlusion: the model should preserve visible local evidence while selectively introducing global semantic information and avoiding misleading contextual associations. The third is geometry-sensitive localization: the localization quality of high-aspect-ratio targets is more sensitive to displacement along the short axis, whereas conventional two-dimensional overlap losses do not explicitly encode this directional sensitivity. These challenges correspond to feature sampling, encoder interaction, and regression supervision, respectively, and are difficult to address through a single local modification.

To address these challenges, we propose Morphology- and Occlusion-Aware Real-Time Detection Transformer (MOR-RTDETR), where MOR denotes morphology enhancement, occlusion-aware feature interaction, and short-axis-aware regression. The model is built on RT-DETR with a ResNet-18 backbone (RT-DETR-R18) and retains the original end-to-end detection pipeline while introducing three complementary modifications. GGME is embedded at the P3 and P4 stages to balance regular-object representation with slender-structure enhancement. BLGIE is inserted at the P5/Attention-based Intra-scale Feature Interaction (AIFI) stage to selectively calibrate and fuse local structural evidence with global semantic information. SAPR is used during training to strengthen short-axis localization for high-aspect-ratio targets. These modifications introduce neither additional detection heads nor post-processing operations and preserve the end-to-end inference behavior of RT-DETR.

Experiments are performed on an existing household gas facility image dataset with a fixed scene-level training–validation split. No additional morphology, curvature, centerline, or instance-level occlusion annotations are introduced. Aspect-ratio subsets are derived automatically from the existing bounding boxes, and image-level degradation tags are used only for auxiliary robustness analysis. The focus of this study is therefore to evaluate the coordinated effectiveness of morphology-aware feature enhancement, local–global interaction, and geometry-sensitive regression, rather than to present a new dataset.

The main contributions of this work are as follows:

1. A GGME module is proposed for mixed-morphology feature representation. GGME combines a standard convolution branch with horizontal and vertical dynamic snake convolution branches and uses fixed feature-gradient cues to guide spatial branch competition, improving the representation of elongated and curved components while preserving conventional texture responses for regular objects.
2. A BLGIE module is proposed for feature interaction under partial visual evidence. BLGIE performs global-to-local channel calibration, local-to-global spatial calibration, and discrepancy-guided reliability fusion, enabling the detector to better combine visible local structures with high-level semantic context without introducing explicit occlusion supervision.
3. A SAPR loss is proposed for geometry-sensitive localization of high-aspect-ratio objects. SAPR is added to the original L1 and GIoU regression objectives and combines short-axis-weighted projected GIoU with elongation-gated shape consistency, improving localization supervision during training without increasing inference-time computation.
4. Extensive experiments on a fixed scene-level household gas facility split evaluate overall detection accuracy, class-wise behavior, component ablations, high-aspect-ratio targets, occlusion-tagged images, and computational efficiency, demonstrating the complementary roles of GGME, BLGIE, and SAPR.

## 2. Materials and Methods

### 2.1. Household Gas Facility Dataset and Task Definition

This study uses a private household gas facility dataset derived from real in-home gas-safety inspection records. The image data were derived from routine household gas-safety inspections rather than collected specifically for research. During the inspections, residents had agreed that staff could photograph on-site equipment for service records and safety assessment. The images were limited to gas-related equipment points, including valves, hoses, pipe clamps, joints, and other relevant facility components. Subsequent research use was conducted with permission from the data controller. Before analysis, all identifying information, including addresses, resident information, timestamps, GPS coordinates, and other metadata, was permanently removed, and privacy-sensitive visual content was excluded, cropped, or blurred where necessary. Therefore, the processed data could not be linked to any specific household or individual. A fixed scene-level split and a unified annotation protocol are used for all experiments. Indoor illumination, camera viewpoint, background texture, appliance layout, and component visibility were not artificially controlled during image acquisition; therefore, the dataset reflects practical residential inspection conditions rather than a laboratory scene.

The images contain multiple on-site disturbances, including cluttered backgrounds, viewpoint variation, partial occlusion, shadows, metallic highlights, oil stains and dust, defocus, and motion blur. These factors often co-occur and increase the difficulty of feature extraction, category discrimination, and bounding-box localization. The fixed split and annotation protocol are used consistently for all baseline, ablation, full-model, and detector-comparison experiments, and this study does not claim a new dataset contribution.

The dataset contains natural variations across household inspection scenes, including differences in appliance layouts, component installation positions, illumination conditions, camera viewpoints, and surrounding backgrounds. These environmental variations are also reflected in the image-level degradation patterns observed in the dataset.. Although the dataset was divided at the household-scene level to reduce data leakage, the present analysis does not quantify the geographical distribution, the number of distinct households, or the distribution of image-acquisition devices. Therefore, the dataset should not be considered geographically or device-balanced, and the reported results are limited to the evaluated residential inspection scenarios.

The detection task is formulated as eight-class horizontal bounding-box object detection. Given an RGB inspection image, the detector is required to predict the category label and horizontal rectangular bounding box for each visible household gas facility component. The task evaluates visual localization of components rather than gas leakage, sealing reliability, component aging, or installation compliance. All target instances were manually annotated by a single annotator using horizontal bounding boxes following a consistent eight-category annotation protocol. Before training and evaluation, the annotations were checked for invalid labels, empty boxes, duplicated boxes, and obvious boundary inconsistencies.

The dataset contains 21,813 images and 47,169 target instances, including 17,450 training images with 37,688 instances and 4363 validation images with 9481 instances. To prevent data leakage, the split was performed at the household-scene level rather than by random image-level partitioning; therefore, images from the same household and near-duplicate frames from continuous acquisition sequences are not shared between the training and validation subsets. Table 1 reports the category-level instance distribution and the main task difficulty associated with each class.

In addition to category imbalance and object-level morphology, the dataset also contains several image-level visual degradations caused by real residential inspection conditions. To support the later robustness analysis, the pre-existing validation image tags are summarized in Table 2. These tags describe complete images rather than individual object instances and are non-exclusive.

Before manuscript visualization, privacy-sensitive header information and household-identifying visual content are removed or cropped when examples are displayed. Because the original images were acquired in private residential environments and may reveal indoor layouts, appliance conditions, and safety-relevant facility configurations, the complete dataset is subject to privacy and data-use restrictions.

### 2.2. Problem Formulation and Baseline

RT-DETR-R18 is used as the principal controlled baseline. Let I denote an input RGB image. The backbone extracts P3, P4, and P5 feature maps. MOR-RTDETR inserts GGME at P3 and P4, replaces the original single AIFI path at P5 with BLGIE while retaining AIFI as its global branch, and applies SAPR only to Hungarian-matched positive boxes during training. The convolutional neural network (CNN)-based Cross-scale Feature Fusion Module (CCFM), query selection, decoder layers, classification loss, auxiliary decoder losses, and the fixed query budget remain unchanged.

{*C*_3_,*C*_4_,*C*_5_} = ℬ(*I*), *P_l_* = *Π_l_*(*C_l_*), *l* ∈ {3,4,5}, (1)

*P*^′^_3_ = *G*(*P*_3_), *P*^′^_4_ = *G*(*P*_4_), *P*^′^_5_ = *L*(*P*_5_), (2)

ℱ*_e_* = {*E*_3_,*E*_4_,*E*_5_} = CCFM(*P*^′^_3_,*P*^′^_4_,*P*^′^_5_), (3)

(4) $$\left(Q_{0},R_{0}\right)=\mathrm{QuerySelect}\left(F_{e},K\right),\;\hat{Y}=\mathrm{Decoder}\left(Q_{0},R_{0},F_{e}\right),$$

**Figure 1:**
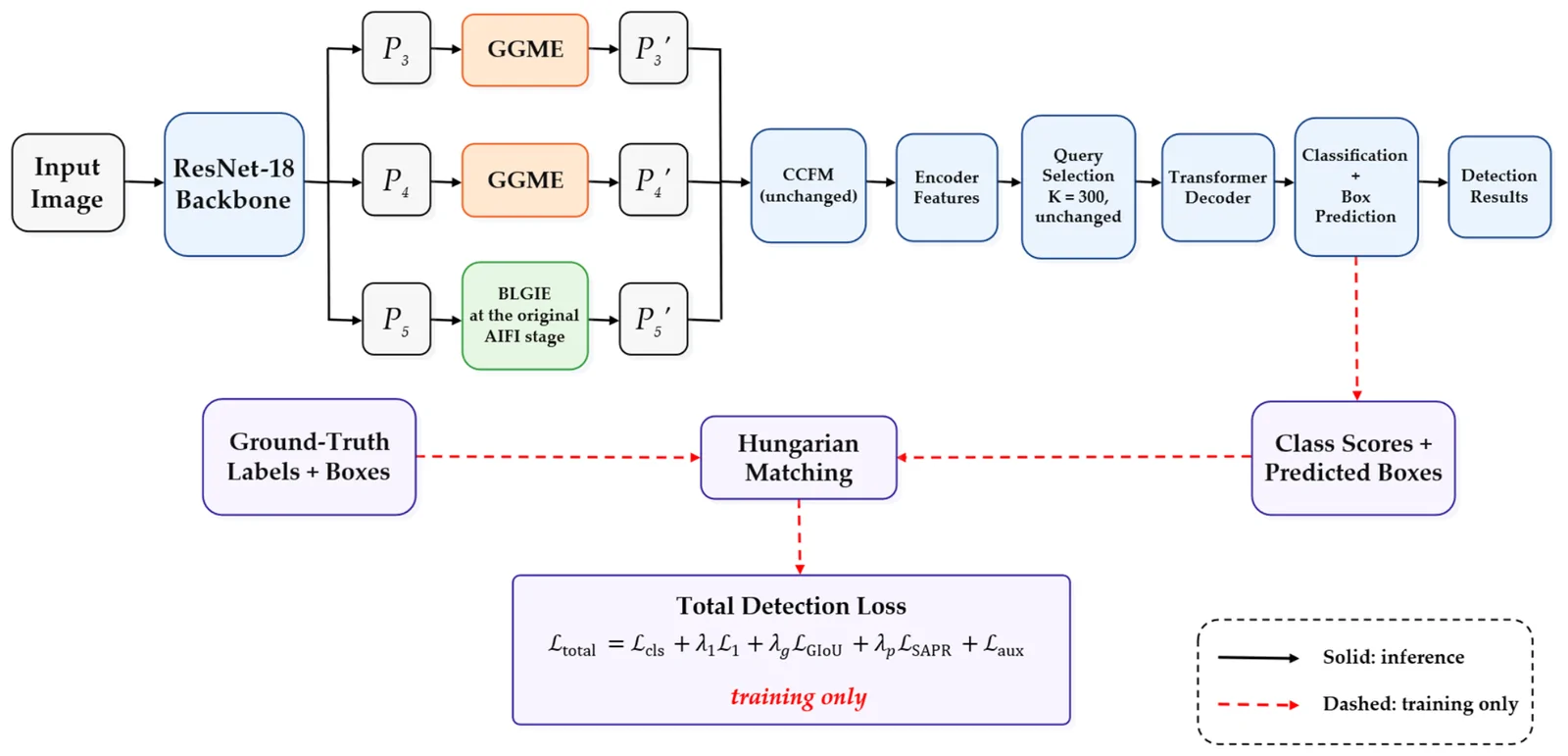
Overall architecture of MOR-RTDETR. GGME is inserted at P3 and P4, BLGIE operates at the original P5/AIFI position, and SAPR is used only during training. The loss notation in the training path is consistent with Equations (45) and (46): ℒ_cls_ denotes the unchanged classification loss, ℒ_1_ and ℒ_GIoU_ denote the original RT-DETR coordinate and GIoU regression terms, ℒ_SAPR_ denotes the proposed training-only short-axis-aware projection loss, and ℒ_aux_ denotes the unchanged auxiliary decoder and denoising losses retained from the official RT-DETR-R18 training configuration. Blue boxes represent the retained RT-DETR components, orange boxes indicate GGME, and the green box indicates BLGIE. Purple-bordered boxes represent supervision-related elements, matching, and loss computation. Solid black arrows indicate the inference pathway, whereas dashed red arrows indicate training-only operations. P3, P4, and P5 denote the backbone feature levels, and the corresponding primed symbols denote the enhanced feature representations.

Here, B denotes the ResNet-18 backbone, and *Π_l_* denotes the feature projection operation at level l. G(·) denotes GGME, L(·) denotes BLGIE, and ℱ*_e_* denotes the multi-scale encoder features produced by the CCFM. $\hat{Y}$ contains class probabilities and normalized horizontal bounding boxes. No NMS or additional inference branch is introduced. The overall architecture of MOR-RTDETR is illustrated in Figure 1.

### 2.3. Gradient-Guided Morphology Enhancement

#### 2.3.1. Multi-Branch Feature Extraction

A fixed 3 × 3 convolution samples features on a regular grid and may therefore incorporate background responses when processing elongated or curved structures such as rubber hoses. DSConv [31] can adapt its sampling trajectory to slender and tortuous structures; however, replacing standard convolution entirely with DSConv may weaken the conventional texture representation required for large or morphologically regular categories. The proposed GGME module therefore retains a standard local branch and introduces two complementary directional DSConv branches.

Let

*F* ∈ ℝ*^B^*^×^*^C^*^×^*^H^*^×^*^W^*, (5)

denote the input feature map, where B, C, H, and W are the batch size, number of channels, height, and width, respectively. A 1 × 1 projection first reduces the channel dimension from C to *C_r_*:

*Z* = *ϕ_r_*(*F*), *C_r_* = *ρC*, (6)

where

(7) $$Z\in R^{B\times C_{r}\times H\times W},$$

*ρ* is the channel-reduction ratio. In the implemented RT-DETR-R18 configuration, the P3 and P4 input features contain 128 and 256 channels, respectively. We set *ρ* = 0.50, producing reduced channel dimensions of 64 at P3 and 128 at P4. The final refinement layer restores the channel dimension to the corresponding input dimension, so the GGME output keeps the same spatial size and channel number as the original P3 or P4 feature and can be passed to the subsequent CCFM without changing the interface. The projected feature Z is then processed in parallel by a standard depthwise-convolution branch and two directional DSConv branches:

*B_s_* = DWConv_3×3_(*Z*), *B_x_* = DSConv*_x_*(*Z*), *B_y_* = DSConv*_y_*(*Z*), (8)

where

(9) $$B_{s},B_{x},B_{y}\in R^{B\times C_{r}\times H\times W},$$

The standard branch Bs preserves conventional local texture and edge information. The directional branches Bx and By adopt horizontally and vertically initialized snake-like sampling trajectories, respectively, and these trajectories are progressively adjusted by learned sequential offsets. Both directional DSConv branches use a kernel length of k = 9. Their offsets are generated by lightweight 3 × 3 convolutions followed by a Tanh activation, which bounds each incremental offset to the range [−1, 1] in feature-grid units before sequential accumulation. Following the topology-preserving design of DSConv [31], the x-direction branch accumulates offsets along the horizontal sampling path, and the y-direction branch accumulates offsets along the vertical sampling path, maintaining trajectory continuity for curved or slender structures. The two directional offset generators are not shared, and no additional offset supervision or offset regularization is introduced. The branches are complementary rather than category-specific, and their contributions are adaptively determined at each spatial position by the gradient-guided branch-competition mechanism described below.

#### 2.3.2. Feature-Gradient Structural Guidance

A branch gate inferred only from learnable branch responses may not consistently associate local structural patterns with the most suitable sampling branch. GGME therefore introduces deterministic feature-gradient descriptors as lightweight structural guidance.

First, channel averaging is applied to the projected feature:

(10) $$Z_{m}={\mathrm{Mean}}_{c}\left(Z\right)=\frac{1}{C_{r}}\sum_{c=1}^{C_{r}}Z_{c},$$

where

*Z_m_* ∈ ℝ*^B^*^×1×^*^H^*^×^*^W^*, (11)

The fixed Sobel kernels are then applied to obtain feature-gradient responses along the horizontal and vertical coordinate axes:

*E_x_* = *K_x_* ∗ *Z_m_*, *E_y_* = *K_y_* ∗ *Z_m_*, (12)

followed by the gradient-magnitude descriptor:

(13) $$E_{m}=\sqrt{E_{x}^{2}+E_{y}^{2}+\varepsilon },$$

where

*E_x_*,*E_y_*,*E_m_* ∈ ℝ*^B^*^×1×^*^H^*^×^*^W^*, (14)

∗ denotes convolution, and *ε* is a small positive constant for numerical stability. The Sobel kernels are fixed and are not updated by backpropagation. The resulting feature-gradient maps are neither treated as ground-truth edges nor supervised by an auxiliary objective; they serve only as deterministic structural cues for branch-weight prediction.

The three branch features and the three gradient descriptors are concatenated along the channel dimension:

*U* = Concat(*B_s_*,*B_x_*,*B_y_*,*E_x_*,*E_y_*,*E_m_*), (15)

where

(16) $$U\in R^{B\times \left(3C_{r}+3\right)\times H\times W},$$

A learnable 1 × 1 convolution *ψ_g_*(·) maps the concatenated representation to three branch logits:

*L* = *ψ_g_*(*U*), *L* ∈ ℝ*^B^*^×3×^*^H^*^×^*^W^*, (17)

A Softmax operation is applied along the three-branch dimension:

[*A_s_*,*A_x_*,*A_y_*] = Softmax(*L*, dim = 1), (18)

where

*A_s_*,*A_x_*,*A_y_* ∈ ℝ*^B^*^×1×^*^H^*^×^*^W^*, (19)

Consequently, for each sample b and spatial position (h, w), the branch weights satisfy:

*A_s_*(*b*,1,*h*,*w*) + *A_x_*(*b*,1,*h*,*w*) + *A_y_*(*b*,1,*h*,*w*) = 1, (20)

The three single-channel weight maps are broadcast along the *C_r_* channel dimension when weighting their corresponding branch features. The morphology-enhanced representation is computed as:

*B_m_* = *A_s_*⊙*B_s_* + *A_x_*⊙*B_x_* + *A_y_*⊙*B_y_*, (21)

where

(22) $$B_{m}\in R^{B\times C_{r}\times H\times W},$$

⊙ denotes element-wise multiplication with channel-wise broadcasting.

Finally, a refinement and channel-restoration operation maps the fused feature from *C_r_* channels back to C channels, after which it is added to the original input through a residual connection:

*F*_out_ = *F* + *ϕ_e_*(*B_m_*), (23)

where

*ϕ_e_*(*B_m_*) ∈ ℝ*^B^*^×^*^C^*^×^*^H^*^×^*^W^*, (24)

This spatial branch-competition mechanism enables GGME to select different sampling behaviors at different feature-map locations while sharing a branch preference across channels at a given position. The standard branch and residual pathway are designed to reduce possible negative transfer to morphologically regular categories.

**Figure 2:**
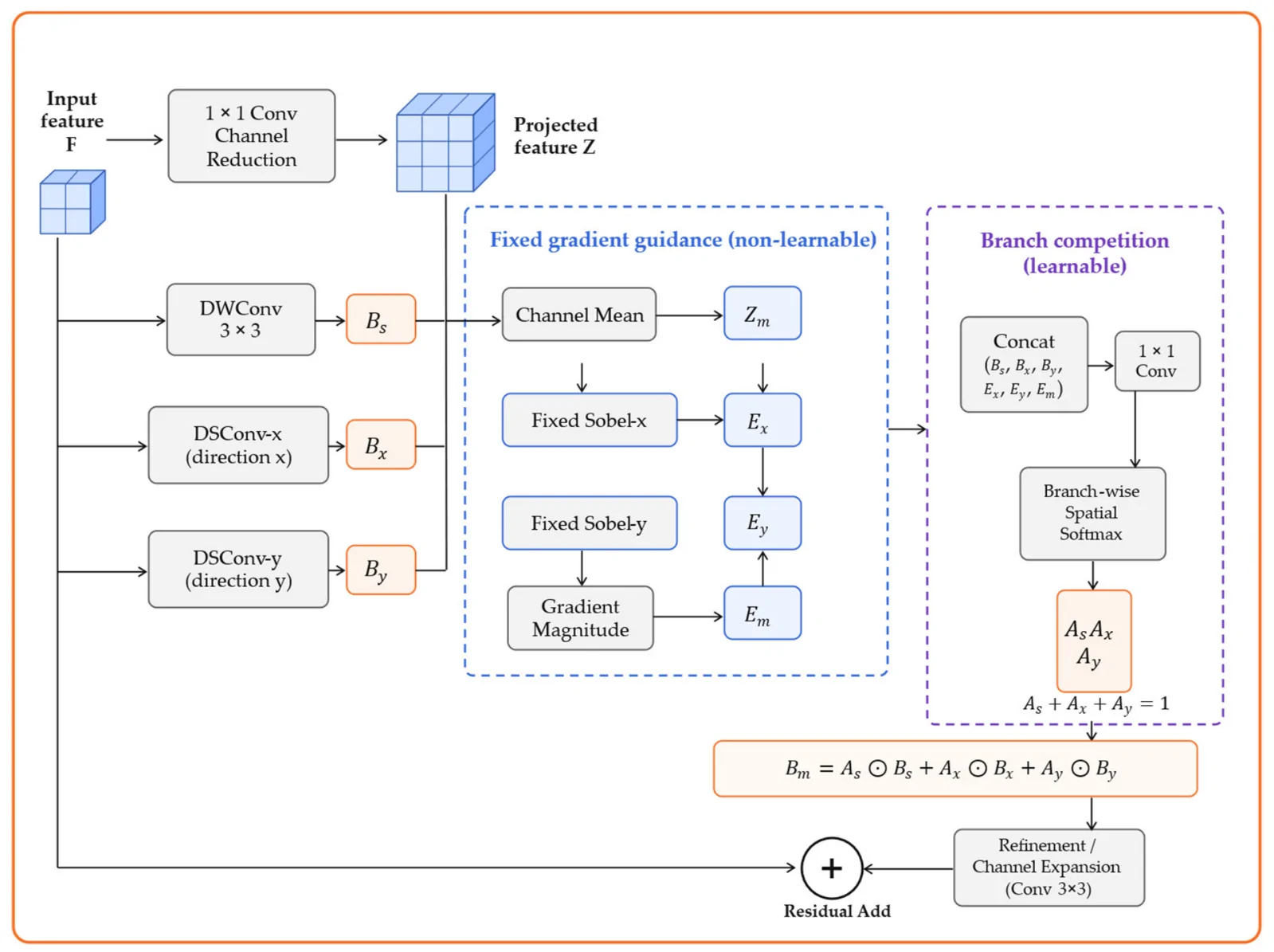
Structure of GGME, including the standard and DSConv branches, fixed Sobel feature-gradient cues, spatial Softmax gate, and residual refinement. Blue and purple dashed boxes indicate the fixed feature-gradient guidance and learnable branch-competition mechanisms, respectively. Solid arrows represent feature processing and information flow, while the circled plus sign denotes residual addition. F and Z denote the input and projected features, respectively. Bs, Bx, and By represent the standard, horizontal DSConv, and vertical DSConv branch features. Zm denotes the channel-averaged feature, while Ex, Ey, and Em represent horizontal, vertical, and magnitude-based gradient descriptors. As, Ax, and Ay are the corresponding spatial branch weights, and Bm denotes the fused feature representation.

GGME is inserted only at the P3 and P4 feature levels. P3 retains relatively fine spatial detail and is important for small or boundary-sensitive components, whereas P4 provides stronger semantic responses while still preserving sufficient local contour information for elongated structures. Inserting GGME at P2 would substantially increase memory use and convolutional cost because of the higher spatial resolution, while inserting it at P5 is less effective for local curvature and narrow structures because the feature map is already strongly downsampled and mainly semantic. The P3/P4 placement therefore balances morphology-adaptive feature sampling, semantic reliability, and computational overhead. The detailed structure of GGME is illustrated in Figure 2.

### 2.4. Bidirectional Local–Global Interaction Encoder

#### 2.4.1. Local and Global Branches

Partial occlusion may remove class-discriminative regions while leaving only visible edges, textures, or component fragments. Local convolutional features are therefore useful for retaining observable structural evidence, whereas AIFI provides complementary long-range semantic information. Given the projected high-level feature:

*X* = *P*_5_ ∈ ℝ*^B^*^×^*^C^*^×^*^H^*^×^*^W^*, (25)

BLGIE constructs parallel local and global branches:

*F_l_* = Conv_1×1_(DWConv_3×3_(LN_2*D*_(*X*))), *F_g_* = AIFI(*X*), (26)

where

*F_l_*,*F_g_* ∈ ℝ*^B^*^×^*^C^*^×^*^H^*^×^*^W^*, (27)

The local branch first applies branch-specific LayerNorm, followed by a 3 × 3 depthwise convolution with padding 1 and a 1 × 1 pointwise convolution for channel interaction. The global branch retains the original AIFI module from the official RT-DETR-R18 configuration to model long-range semantic dependencies. The normalization layers of the local and global branches are not shared. Because the global branch keeps the original AIFI structure, the pretrained AIFI-related weights and surrounding projection weights can be inherited from the RT-DETR-R18 checkpoint, whereas the newly introduced local, calibration, and fusion layers are randomly initialized and learned under the same detection supervision.

#### 2.4.2. Bidirectional Cross-Calibration

Direct addition or concatenation of local and global features does not explicitly model their complementary roles. BLGIE therefore introduces two asymmetric calibration paths.

First, global semantic information generates channel-wise weights to recalibrate the local representation:

*c_g_* = *σ*[*W*_2_*δ*(*W*_1_GAP(*F_g_*))], *F_lc_* = *F_l_*⊙*c_g_*, (28)

where

*c_g_* ∈ ℝ*^B^*^×^*^C^*^×1×1^, (29)

is broadcast along the spatial dimensions.

Conversely, local structural evidence generates a spatial weight map to recalibrate the global representation:

*s_l_* = *σ*{Conv_3×3_[Avg*_c_*(*F_l_*); Max*_c_*(*F_l_*)]}, *F_gs_* = *F_g_*⊙*s_l_*, (30)

where

*s_l_* ∈ ℝ*^B^*^×1×^*^H^*^×^*^W^*, (31)

is broadcast along the channel dimension.

The two calibration directions are intentionally asymmetric. Global semantic information is used for channel calibration because different channels encode different semantic responses, whereas local evidence is used for spatial calibration because visible fragments provide location-dependent structural cues. In the global-to-local calibration path, global average pooling (GAP) is followed by a bottleneck multilayer perceptron (MLP) with a reduction ratio of 4 and a Sigmoid activation to generate channel-wise weights. In the local-to-global calibration path, channel-average and channel-maximum descriptors are concatenated and processed by a 3 × 3 convolution followed by a Sigmoid activation to generate the spatial calibration map. This design keeps the calibration lightweight while explicitly modeling the different reliability patterns of local structure and global semantics.

#### 2.4.3. Discrepancy-Guided Reliability Fusion

After cross-calibration, the absolute discrepancy between the local and global representations is used as an additional reliability cue:

*D* = ∣*F_lc_* − *F_gs_*∣, *Q* = *σ*[Conv_1×1_([*F_lc_*; *F_gs_*; *D*])], (32)

In the recommended channel-spatial implementation:

*D*, *Q* ∈ ℝ*^B^*^×^*^C^*^×^*^H^*^×^*^W^*, (33)

The final fused representation is obtained as:

*F*_out_ = *X* + *ϕ_o_*[*Q*⊙*F_lc_* + (1 − *Q*)⊙*F_gs_*], (34)

where *φ_o_*(·) denotes a 1 × 1 projection or refinement operation, and:

*F*_out_ ∈ ℝ*^B^*^×^*^C^*^×^*^H^*^×^*^W^*, (35)

**Figure 3:**
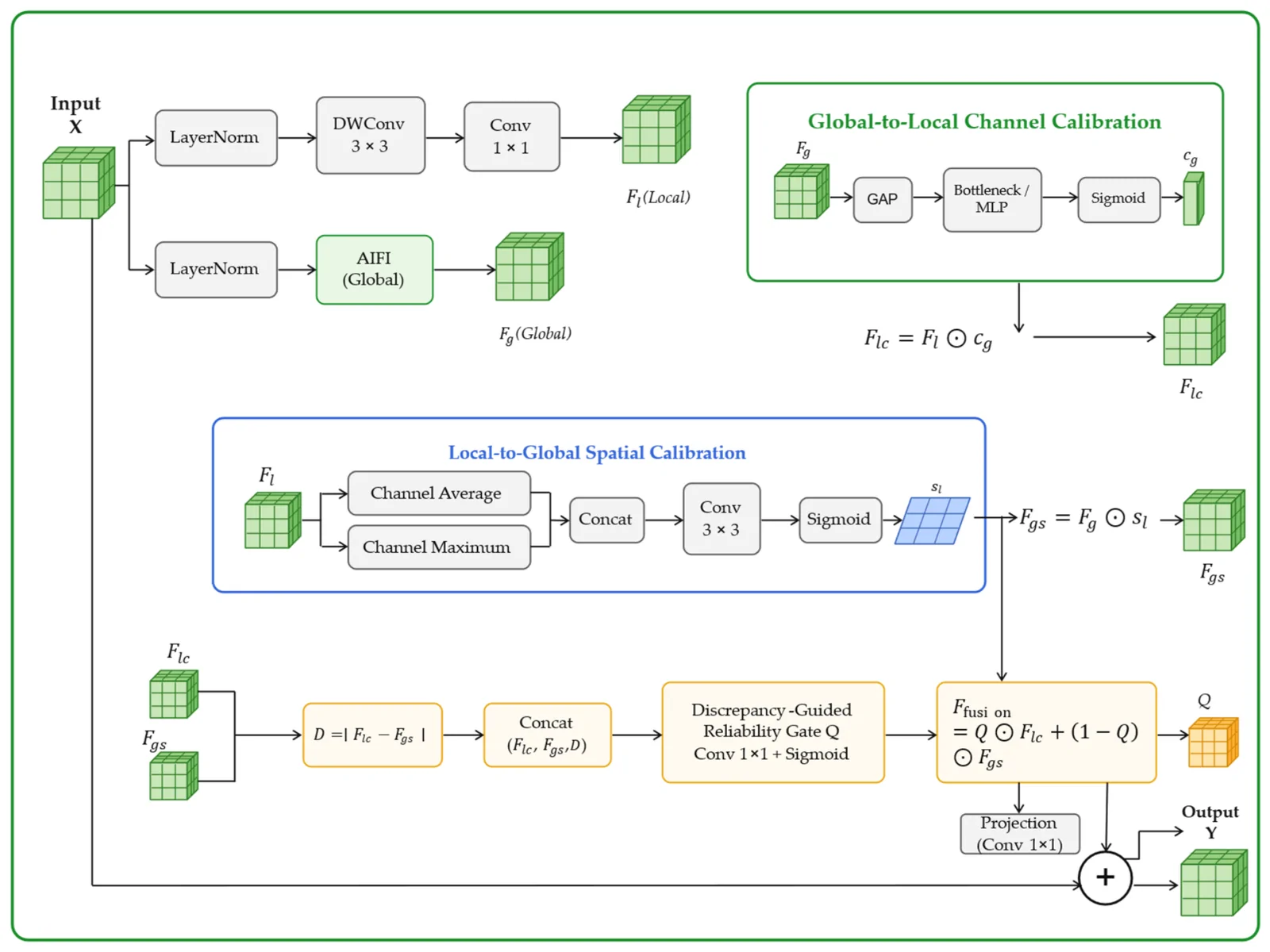
Structure of BLGIE. The module constructs local and global branches, performs global-to-local channel calibration and local-to-global spatial calibration, and fuses the calibrated representations using a discrepancy-guided reliability gate. Green feature blocks represent intermediate feature maps. The green- and blue-outlined regions indicate global-to-local channel calibration and local-to-global spatial calibration, respectively, while orange-outlined blocks represent discrepancy computation and reliability-guided fusion. Solid arrows indicate information flow, and the circled plus sign denotes residual addition. X and Y denote the input and output features; F_l_ and F_g_ denote the local and global features; c_g_ and s_l_ represent channel and spatial calibration weights; F_lc_ and F_gs_ denote the calibrated features; and D and Q represent feature discrepancy and the learned reliability gate, respectively. GAP and MLP denote global average pooling and multilayer perceptron, respectively.

The learned gate Q estimates the relative reliability of the calibrated local and global representations at different channel-spatial positions. It should not be interpreted as an explicit occlusion map or occlusion probability, because BLGIE introduces neither pixel-level occlusion annotations nor an auxiliary occlusion-supervision objective. Instead, BLGIE is used as a reliability-guided local–global feature fusion module. Its effect under partial visual evidence is evaluated on the pre-existing occlusion-tagged validation subset, which contains 1255 images and corresponds to 28.77% of the validation set; these tags are image-level and non-exclusive rather than instance-level occlusion annotations. Because these images may also contain blur, reflection, stains, low illumination, or background clutter, the occlusion-tag AP is interpreted as diagnostic evidence of improved detection on images containing partial visual evidence, not as proof of instance-level occlusion localization or isolated causal robustness to occlusion. The complete BLGIE structure is summarized in Figure 3.

### 2.5. Short-Axis-Aware Projection Regression Loss

#### 2.5.1. Motivation

For an elongated ground-truth box, the same absolute localization displacement along the short axis corresponds to a larger relative error than displacement along the long axis and may therefore cause a more pronounced decrease in bounding-box overlap. For example, a small vertical displacement of a horizontally elongated rubber hose can substantially reduce its IoU even when the horizontal extent is accurately estimated. Conventional two-dimensional IoU-based losses jointly measure alignment along the two coordinate axes but do not explicitly emphasize the axis that is more sensitive to localization errors.

**Figure 4:**
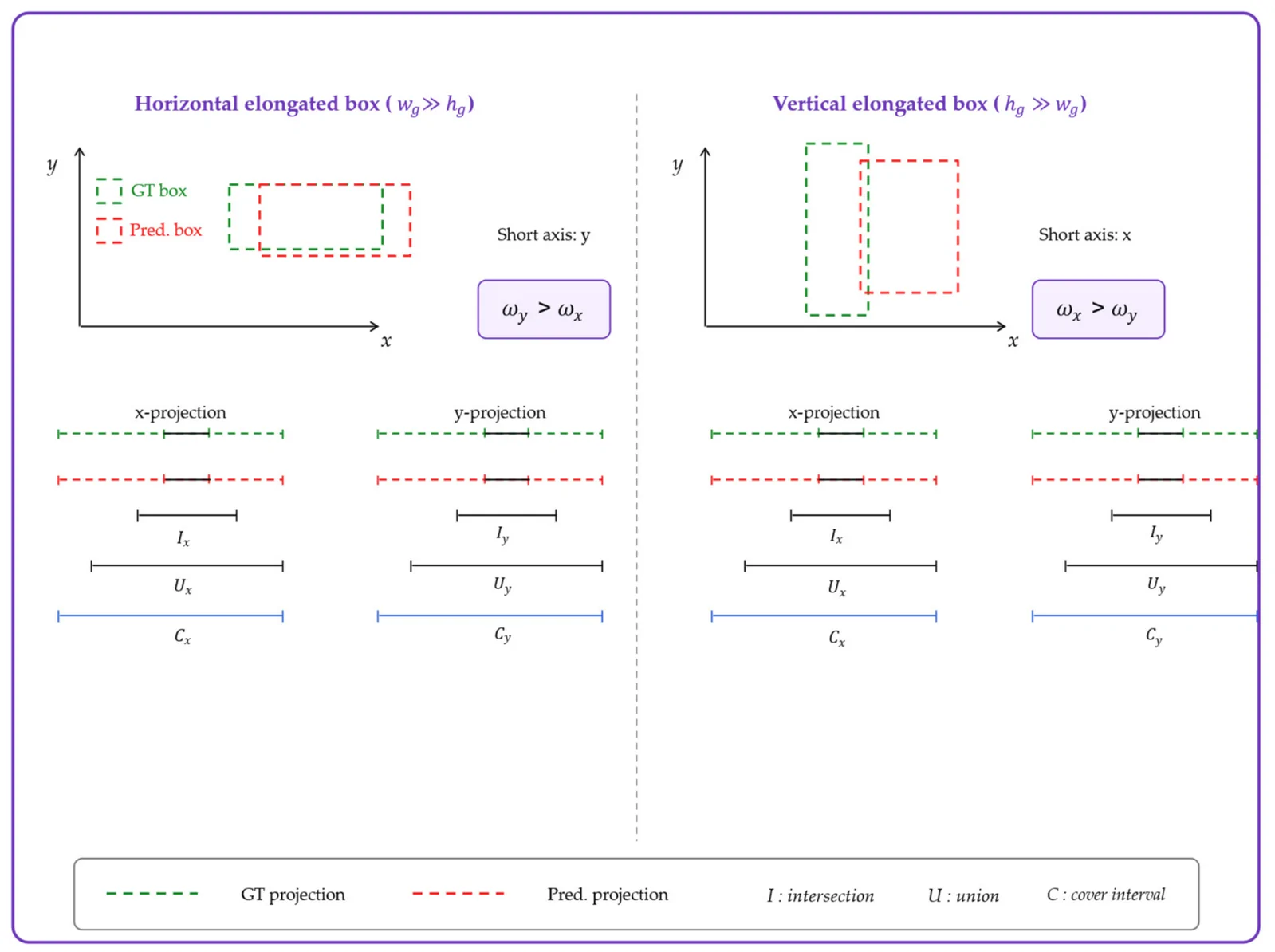
Geometric motivation of SAPR. For high-aspect-ratio horizontal boxes, a displacement along the short axis causes a disproportionately sensitive projection misalignment. The same principle applies to vertical elongated boxes, motivating short-axis-weighted projected regression during training. Black horizontal line segments represent the intersection (I) and union (U) intervals, while blue horizontal line segments represent the smallest enclosing intervals (C) of the projected boxes.

To address this limitation, SAPR decomposes bounding-box alignment into one-dimensional projections along the x- and y-axes. The projection corresponding to the ground-truth short axis is assigned a larger weight, and an elongation-gated shape-consistency term further regularizes the predicted aspect ratio. SAPR is added to the original L1 and GIoU objectives rather than replacing them, thereby retaining general two-dimensional geometric supervision, particularly for non-overlapping boxes and morphologically regular objects. The geometric motivation is illustrated in Figure 4, which shows how the short-axis projection receives greater emphasis for both horizontally and vertically elongated boxes.

#### 2.5.2. One-Dimensional Projected GIoU

For a Hungarian-matched positive pair, let the predicted box and the ground-truth box be denoted by:

(36) $$b^{p}=\left(x_{l}^{p},y_{t}^{p},x_{r}^{p},y_{b}^{p}\right),\;b^{g}=\left(x_{l}^{g},y_{t}^{g},x_{r}^{g},y_{b}^{g}\right),$$

For each projection axis a ∈ {x, y}, let *I_a_*, *U_a_*, and *C_a_* denote the lengths of the intersection interval, union interval, and smallest enclosing interval, respectively. The one-dimensional projected GIoU is defined as:

(37) $$G_{a}=\frac{I_{a}}{U_{a}+\varepsilon }-\frac{C_{a}-U_{a}}{C_{a}+\varepsilon },\;a\in \{x,y\},$$

where *ε* is a small positive constant for numerical stability.

For the x-axis, the three interval lengths are obtained from the projected x-coordinate intervals; the y-axis quantities are defined analogously from the projected y-coordinate intervals. Unlike ordinary one-dimensional IoU, projected GIoU retains an enclosing-interval penalty when the corresponding projected intervals do not overlap.

#### 2.5.3. Short-Axis Weighting and Shape Consistency

Let *w_g_* and *h_g_* denote the width and height of the ground-truth box. The projection weights are derived from the opposite side lengths so that the projection along the short axis receives a larger weight:

(38) $$\omega_{x}=\frac{{\left(h_{g}+\varepsilon \right)}^{\gamma }}{{\left(w_{g}+\varepsilon \right)}^{\gamma }+{\left(h_{g}+\varepsilon \right)}^{\gamma }},\;\omega_{y}=\frac{{\left(w_{g}+\varepsilon \right)}^{\gamma }}{{\left(w_{g}+\varepsilon \right)}^{\gamma }+{\left(h_{g}+\varepsilon \right)}^{\gamma }},$$

where *γ* > 0 controls the sensitivity of the weighting function to the ground-truth aspect ratio, and:

*ω_x_* + *ω_y_* = 1, (39)

For a horizontally elongated target, the vertical projection receives a larger weight and therefore emphasizes alignment along the short-axis direction. Conversely, for a vertically elongated target, the horizontal projection becomes dominant. The weighted projection loss is defined as:

ℒ_proj_ = *ω_x_*(1 − *G_x_*) + *ω_y_*(1 − *G_y_*), (40)

To further constrain shape estimation for elongated targets, an elongation-gated aspect-ratio consistency term is introduced:

(41) $$e_{g}=\tanh(|\ln\frac{w_{g}+\varepsilon }{h_{g}+\varepsilon }|),$$

(42) $$\Delta_{r}=|\ln\frac{w_{p}+\varepsilon }{h_{p}+\varepsilon }-\ln\frac{w_{g}+\varepsilon }{h_{g}+\varepsilon }|,$$

ℒ_shape_ = *e_g_*SmoothL1(Δ*_r_*), (43)

where *w_p_* and *h_p_* denote the predicted width and height.

The elongation factor approaches zero for approximately square ground-truth boxes and increases with elongation. Consequently, the shape-consistency term has limited influence on regular objects while imposing stronger aspect-ratio supervision on elongated targets.

#### 2.5.4. Complete Training Objective

SAPR combines the short-axis-aware projection term and the elongation-gated shape term:

ℒ_SAPR_ = ℒ_proj_ + *λ_s_*ℒ_shape_, (44)

where *λ_s_* controls the contribution of the shape-consistency term. The complete bounding-box regression objective is:

ℒ_box_ = *λ*_1_ℒ_1_ + *λ_g_*ℒ_GIoU_ + *λ_p_*ℒ_SAPR_, (45)

where the weighting coefficients balance the three regression terms.

The overall training objective is expressed as:

ℒ_total_ = ℒ_cls_ + ℒ_box_ + ℒ_aux_, (46)

where the classification loss and auxiliary decoder losses are kept unchanged from the baseline. In our implementation, the official RT-DETR-R18 denoising branch is retained with its default configuration; its loss is included in ℒ_aux_ and is kept unchanged for all baseline, ablation, and full-model experiments.

**Figure 5:**
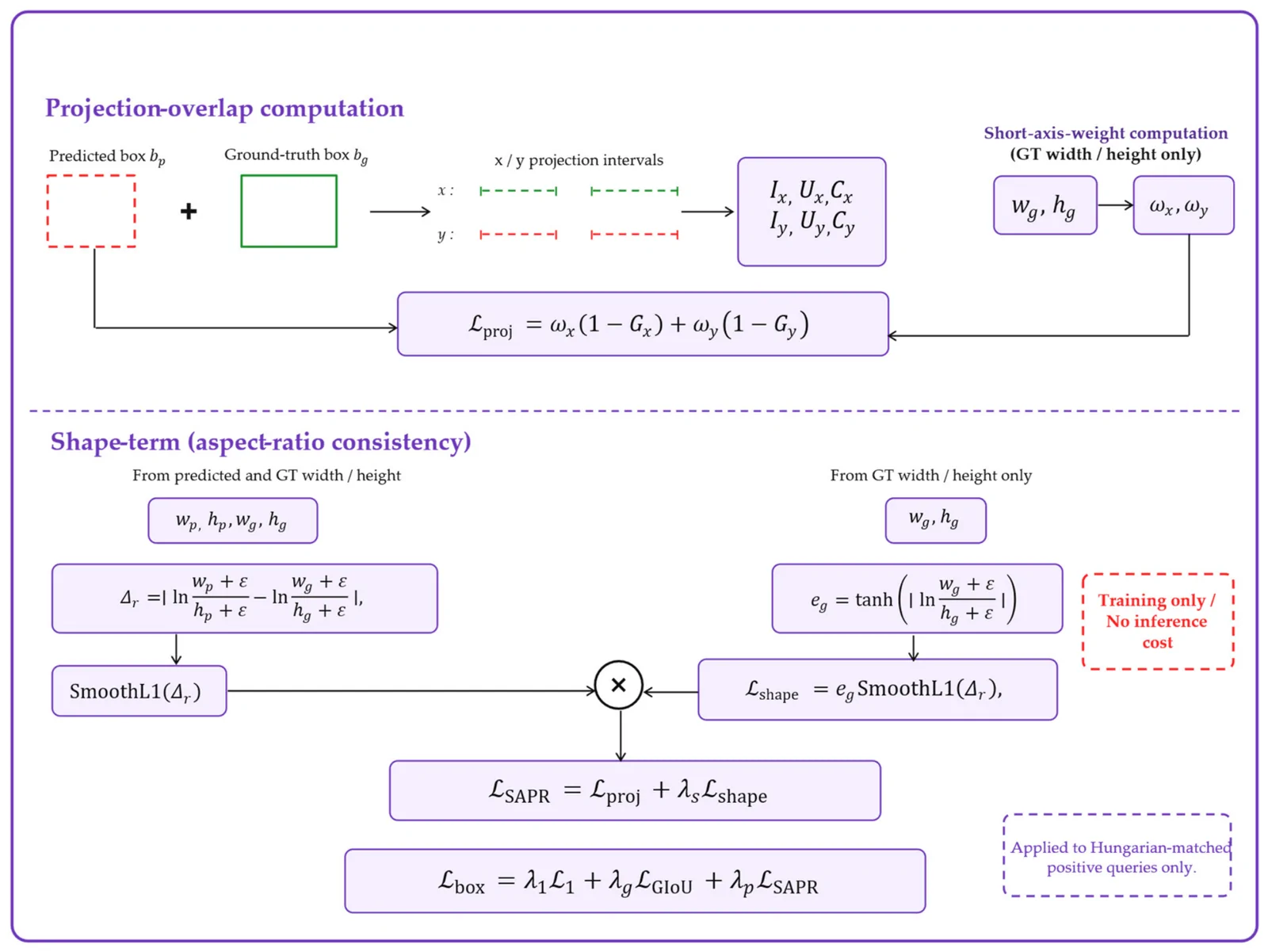
SAPR loss composition. The projection-overlap term computes one-dimensional projected GIoU along the x- and y-axes, the short-axis weights are derived from the ground-truth width and height, and the elongation-gated shape term is applied only during training. The notation follows Equations (44) and (45), where the SAPR loss combines the projection loss with the shape-consistency loss weighted by *λ_s_* and is added to the original ℒ_1_ and GIoU regression objectives. The circled multiplication symbol (×) denotes the multiplication of the elongation factor and the SmoothL1 aspect-ratio discrepancy term. The red dashed box and green solid box represent the predicted and ground-truth bounding boxes, respectively, while the corresponding dashed horizontal lines indicate their projections along the x- and y-axes. Purple-outlined boxes represent intermediate computations and loss terms, solid arrows indicate the computational flow, and dashed annotations highlight training-only operations and their application conditions.

SAPR is evaluated only for Hungarian-matched positive box pairs and is not included in the Hungarian matching cost. The original classification, L1, and GIoU matching costs are retained so that the effect of SAPR can be attributed to regression supervision rather than to changes in assignment strategy. Because SAPR is used only during training, it introduces no additional inference-time parameters or operations. The overall SAPR loss composition is illustrated in Figure 5.

### 2.6. Evaluation Subsets Derived Without New Annotation

Targeted evaluations are conducted using subsets derived automatically from existing bounding-box annotations and previously available image-level degradation tags. No new morphology, scale, or occlusion annotations are introduced.

For scale-specific evaluation, the standard Microsoft Common Objects in Context (COCO) area ranges are adopted following COCO evaluation conventions [42]. Let:

*a_g_* = *w_g_h_g_*, (47)

denote the area of a ground-truth box in the original annotation coordinate system. The scale subsets are defined as:

Small, *a_g_* < 32^2^, Medium, 32^2^ ≤ *a_g_* < 96^2^, Large, *a_g_* ≥ 96^2^. (48)

Scale-specific metrics are computed using the standard COCO evaluation protocol. Ground-truth objects outside the selected area range are treated as ignored instances rather than removed from the annotation set. This avoids incorrectly counting detections of out-of-range objects as false positives. The official COCO evaluator similarly uses area ranges to determine which ground-truth objects and unmatched detections are ignored during scale-specific evaluation.

Aspect-ratio subsets are derived from each ground-truth horizontal bounding box using:

(49) $$r_{g}=\max\left(\frac{w_{g}}{h_{g}},\frac{h_{g}}{w_{g}}\right),r_{g}\geq 1,$$

The validation instances are divided into three mutually exclusive groups:

Regular, 1 ≤ *r_g_* < 2, Moderately elongated, 2 ≤ *r_g_* < 4, Highly elongated, *r_g_* ≥ 4, (50)

These fixed intervals are used as mechanism-oriented diagnostic groups rather than as new object attributes. Regular-aspect-ratio objects provide a control for possible negative transfer, whereas highly elongated objects constitute the primary evaluation group for GGME and SAPR. Aspect-ratio subsets are derived automatically from horizontal bounding-box annotations and are used only for diagnostic evaluation of elongated-object localization.

When aspect-ratio-stratified average precision (AP) is calculated, the same prediction set is retained for all groups. Ground-truth instances outside the selected aspect-ratio interval are marked as ignored rather than deleted. This prevents valid predictions corresponding to non-target aspect-ratio objects from being incorrectly counted as false positives.

For secondary robustness evaluation, the fixed validation images are additionally grouped using the pre-existing, non-exclusive image-level degradation tags listed in Table 2: blur, reflection or overexposure, stain or dust, occlusion, low illumination or shadow, and background clutter. In this study, quantitative degradation-subset analysis focuses on the occlusion-tagged images, while the remaining image-level tags are used to characterize the visual diversity of the dataset.

Because these tags describe complete images rather than individual object instances, the resulting metrics reflect performance on images containing a particular degradation condition. They are not interpreted as instance-level measurements of occlusion severity or as causal estimates of the effect of a single degradation. An image may also belong to more than one degradation subset.

All evaluation subsets are therefore generated from existing annotations or previously available image-level metadata, and no additional manual attribute annotation is required.

### 2.7. Implementation Details

For the controlled experiments involving RT-DETR-R18, MOR-RTDETR, and the corresponding ablation variants, the same scene-level training–validation split, annotation format, input resolution, training schedule, data augmentation policy, and evaluation protocol were used. Unless otherwise stated, only the explicitly specified module or loss term was modified in each controlled experiment. The corresponding training configuration is summarized in Table 3.

For the mainstream detector comparisons, all models used the same fixed household-scene-level training–validation split, eight-category annotation scheme, 640 × 640 input resolution, and COCO-style evaluation protocol. However, identical optimization hyperparameters were not imposed across different detection architectures. Instead, architecture-specific training configurations were adopted and documented to accommodate their different optimization characteristics. In contrast, the controlled comparisons between RT-DETR-R18 and MOR-RTDETR, including the corresponding ablation variants, employed the same optimizer, learning-rate schedule, batch size, data augmentation policy, maximum training epochs, and checkpoint-selection protocol. Therefore, the statement regarding identical training settings applies specifically to the controlled RT-DETR-based experiments rather than to all mainstream detectors. The shared configuration of RT-DETR-R18 and MOR-RTDETR is provided in Table 3, while the implementation details of the competing detectors are presented in Section 3.3. 

During training, the fixed validation set containing 4363 images was used to monitor detection performance. For each independent run, the checkpoint achieving the highest validation COCO-style mAP@[0.50:0.95] was saved as the best-performing checkpoint. The selected checkpoint was subsequently used to report detection performance on the same validation split. The maximum training duration was set to 300 epochs, with an early-stopping patience of 15 epochs. Accordingly, the actual number of training epochs could be lower than the specified maximum, and the best-performing checkpoint was selected according to validation mAP@[0.50:0.95]. Therefore, the reported accuracy results represent performance on the model-selection validation set rather than on an independent test set.

For the experiments requiring seed-wise reporting, three independent runs were conducted using random seeds 42, 123, and 2024. The random seeds were set for Python’s random module, NumPy, PyTorch CPU and CUDA random number generators, and DataLoader worker initialization.

The task-specific module hyperparameters were kept fixed across the relevant ablation and full-model experiments. For GGME, the channel-reduction ratio is *ρ* = 0.50, the DSConv kernel length is k = 9, and the offset increments are bounded by Tanh before sequential accumulation. For BLGIE, the local branch uses a 3 × 3 depthwise convolution and a 1 × 1 pointwise convolution; the global branch uses the official RT-DETR-R18 AIFI structure; and the channel-calibration MLP reduction ratio is 4. For SAPR, the regression weights are *λ*_1_ = 5.0, *λ_g_* = 2.0, *λ_p_* = 1.0, and *λ*_s_ = 0.50; the short-axis weighting sensitivity is *γ* = 1.5, and the numerical-stability constant is *ε* = 1 × 10^−7^. SAPR is applied only to Hungarian-matched positive boxes and is excluded from the Hungarian matching cost, so it introduces no additional inference-time parameters or operations. The sensitivity of the five representative architectural and loss-related hyperparameters was further evaluated through controlled experiments, as described in Section 3.5.3.

## 3. Results

### 3.1. Evaluation Protocol and Statistical Reporting

The primary metric is COCO-style mean average precision (mAP)@[0.50:0.95]. Secondary metrics include AP at an IoU threshold of 0.50 (AP50), AP at an IoU threshold of 0.75 (AP75), AP for small objects (APsmall), class-wise AP, aspect-ratio-stratified AP, and AP on the pre-existing degradation-tagged image lists. Unless otherwise stated, accuracy metrics in the main comparison and key ablation tables are reported as mean ± standard deviation over three independent runs using seeds 42, 123, and 2024. Parameter count and floating-point operations (FLOPs) are deterministic at 640 × 640 input resolution, whereas frames per second (FPS) is measured under the unified batch-1 FP32 protocol specified in Table 3.

For inference efficiency evaluation, all detectors were benchmarked on the same NVIDIA GeForce RTX 4060 Laptop GPU (8 GB) at an input resolution of 640 × 640, using batch size 1 and FP32 precision. After 50 warm-up iterations, inference latency was measured over 200 timed iterations, including image preprocessing, model inference, and post-processing. CUDA synchronization was performed at the timing boundaries to account for asynchronous GPU execution. FPS was calculated by dividing the number of processed images by the total measured elapsed time. Because the same validation split was used for checkpoint selection and final performance reporting, the reported results should be interpreted as within-dataset validation performance rather than independent test-set estimates.

### 3.2. Overall Performance Comparison

Table 4 compares MOR-RTDETR with the RT-DETR-R18 baseline under the same fixed validation split and three-seed protocol. MOR-RTDETR increases COCO-style mAP from 0.6767 to 0.7108, AP50 from 0.8543 to 0.8782, AP75 from 0.7449 to 0.7987, and APsmall from 0.4060 to 0.4928. The absolute gains are therefore 3.41 percentage points in mAP, 2.39 points in AP50, 5.38 points in AP75, and 8.68 points in APsmall. The model adds 2.4 M parameters and 5.9 giga floating-point operations (GFLOPs), while reducing throughput from 76.2 to 72.3 FPS. The larger improvements in AP75 and APsmall are consistent with the intended emphasis on geometry-sensitive localization and weak-response compact components.

The observed performance gains are also substantial relative to the run-to-run variability across the three random seeds. For mAP, the mean improvement is 0.0341, compared with standard deviations of 0.0029 and 0.0026 for RT-DETR-R18 and MOR-RTDETR, respectively. Similarly, the improvements in AP50, AP75, and APsmall exceed the corresponding standard deviations reported in Table 4. These results suggest that the observed gains are relatively stable under the present experimental protocol. Nevertheless, the limited number of independent runs restricts the strength of statistical inference, and no formal claim of statistical significance is made.

### 3.3. Comparison with Mainstream Detectors

To evaluate the proposed method against representative object detection architectures, several mainstream detectors were selected for comparison. Faster R-CNN-R50 [7] represents the conventional two-stage detection framework, while YOLOv8n/s [43] and YOLOv10n/s [44] represent lightweight real-time detectors with different model capacities. Deformable DETR-R50 [21] and DINO-R50 [22] provide Transformer-based detection references, whereas RT-DETR-R18 [23] serves as the direct architectural baseline of MOR-RTDETR. These models were selected to cover different detection paradigms and accuracy–efficiency trade-offs. Their implementation sources and training configurations are summarized in Table 5.

The cross-architecture comparisons were intended to assess the relative performance of representative detector systems under a common dataset and evaluation protocol, rather than to isolate architectural differences under identical optimization settings. In contrast, RT-DETR-R18 and MOR-RTDETR were directly compared under matched training configurations, allowing the incremental effect of the proposed modifications to be assessed more consistently.

Table 6 presents a quantitative comparison of representative object detectors on the household gas facility dataset under the same scene-level data split, input resolution, evaluation metrics, and inference protocol. As detailed in Table 5, the comparison detectors generally follow their respective architecture-specific training configurations. To further examine the influence of training duration, YOLOv10s was additionally evaluated using the same maximum training budget of 300 epochs and early-stopping patience of 15 epochs as RT-DETR-R18 and MOR-RTDETR, while retaining architecture-specific optimization settings. Under the evaluated conditions, MOR-RTDETR achieves the highest mAP (0.711), AP75 (0.799), and APsmall (0.493) among the compared detectors. Compared with YOLOv8s, its mAP, AP75, and APsmall increase by 4.7, 6.7, and 8.0 percentage points, respectively. Compared with DINO-R50, the corresponding improvements are 2.9, 4.9, and 6.0 percentage points. In terms of inference efficiency, MOR-RTDETR achieves 72.3 FPS, compared with 112.7 FPS for YOLOv10s and 11.8 FPS for DINO-R50. These results indicate that MOR-RTDETR provides improved localization accuracy, particularly for small objects, while maintaining real-time inference capability. The observed advantages are specific to the evaluated residential inspection scenarios and should be interpreted alongside the differences in model architectures and training configurations.

RT-DETR-R18 was selected as the baseline architecture because its multi-scale P3/P4 features, attention-based intra-scale feature interaction (AIFI) at P5, and end-to-end detection framework provide well-defined integration points for the proposed modifications. Specifically, GGME enhances the P3 and P4 feature representations, BLGIE strengthens global–local contextual interaction at the P5 AIFI stage, and SAPR introduces short-axis-aware regression supervision during training without modifying the inference procedure. Unlike the dense-prediction and NMS-based pipeline of YOLOv8, RT-DETR employs query-based end-to-end detection, whereas YOLOv10 adopts a different NMS-free detection framework. These architectural differences mean that transferring the proposed modules to YOLO would require adaptation to the corresponding feature-processing and regression mechanisms, and such transferability is not established by the present experiments. Nevertheless, the controlled comparison with RT-DETR-R18 in Table 4 demonstrates that MOR-RTDETR improves mAP from 0.6767 to 0.7108 (+3.41 percentage points), AP75 from 0.7449 to 0.7987 (+5.38 percentage points), and APsmall from 0.4060 to 0.4928 (+8.68 percentage points). Furthermore, compared with the YOLOv10s model trained under the matched maximum-epoch setting in Table 6, MOR-RTDETR achieves higher mAP (0.7108 versus 0.6863) and AP75 (0.7987 versus 0.7578), while exhibiting lower inference throughput (72.3 versus 112.7 FPS). These findings quantitatively demonstrate the accuracy–efficiency trade-off and the benefits of the proposed design within the evaluated residential inspection scenarios.

**Figure 6:**
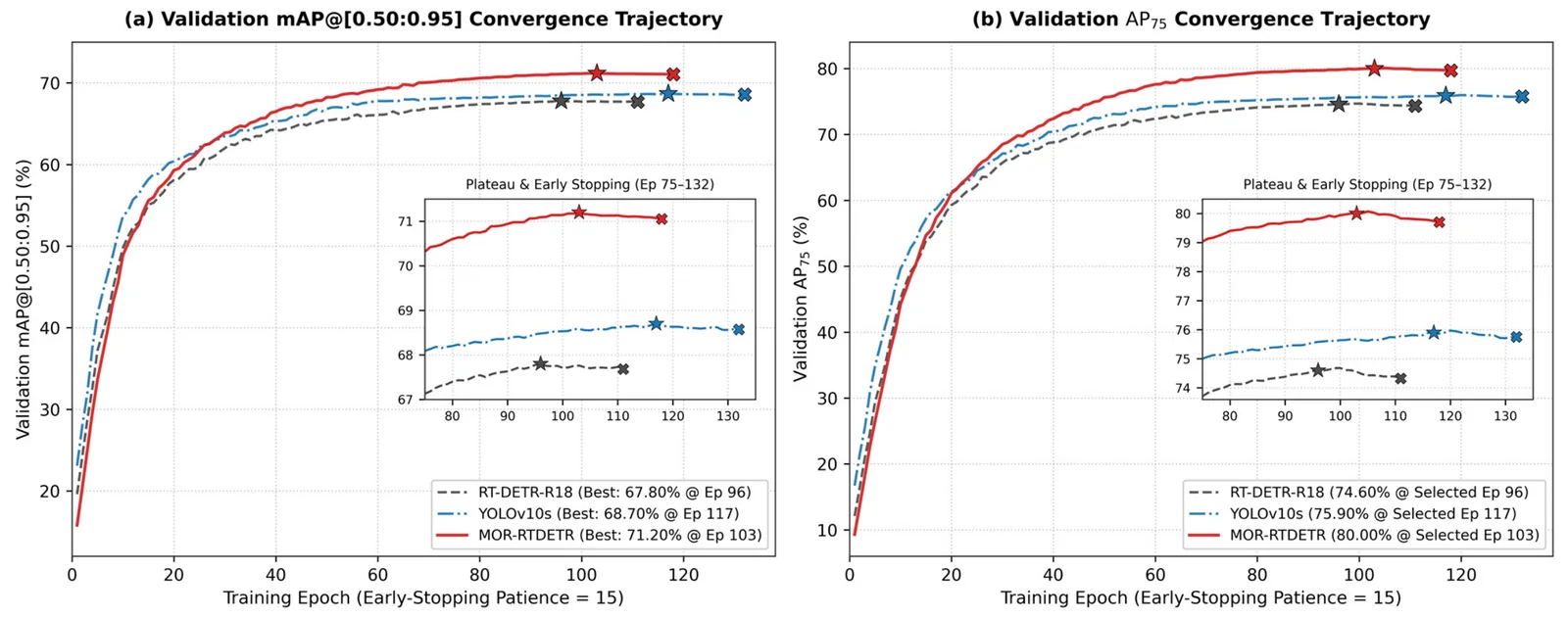
Validation convergence comparison of RT-DETR-R18, YOLOv10s, and MOR-RTDETR under a matched maximum training budget of 300 epochs with an early-stopping patience of 15 epochs. (**a**) Validation mAP@[0.50:0.95] versus training epoch. (**b**) Validation AP75 versus training epoch. Curves correspond to random seed 42. Star markers indicate checkpoints selected according to the highest validation mAP, while cross markers indicate the actual early-stopping epochs. AP75 values at the selected checkpoints are reported in the legend.

To further assess the influence of training duration, we compared the validation convergence of RT-DETR-R18, YOLOv10s, and MOR-RTDETR under a common maximum training budget of 300 epochs and an early-stopping patience of 15 epochs. All models used the same scene-level data split and evaluation protocol, while retaining their architecture-specific optimization configurations. Figure 6 presents the epoch-wise validation mAP@[0.50:0.95] and AP75 trajectories for the representative run with random seed 42. The best-performing checkpoints were selected according to validation mAP@[0.50:0.95].

As illustrated in Figure 6, the three representative detectors exhibit increasing validation performance followed by convergence before reaching the maximum training limit of 300 epochs. For random seed 42, RT-DETR-R18, YOLOv10s, and MOR-RTDETR achieve their best validation mAP at epochs 96, 117, and 103, respectively, and training terminates at epochs 111, 132, and 118 under the early-stopping criterion. The corresponding selected-checkpoint mAP values are 67.80%, 68.70%, and 71.20%, while the AP75 values are 74.60%, 75.90%, and 80.00%, respectively. These results show that MOR-RTDETR retains an accuracy advantage over the two representative comparison models under the matched maximum-epoch setting. The results also indicate that the observed performance differences cannot be attributed solely to a higher maximum epoch limit, although the architecture-specific optimization settings remain different.

### 3.4. Class-Wise Performance and Error Diagnosis

Table 7 shows that MOR-RTDETR improves AP across all eight categories. The gains are modest for visually salient regular objects such as Water_heater, Stove, and Flue, whereas larger gains are observed for Pipe_clamp, Ft_protection, Valve, and Rubber_hose. This pattern indicates that the proposed morphology enhancement, local–global interaction, and short-axis-aware regression mainly benefit compact, elongated, context-dependent, and boundary-sensitive components while preserving performance on regular categories. Pipe_clamp achieves the largest gain because its small size, weak contour, and adjacency to pipes make it sensitive to morphology-aware sampling and stricter boundary supervision. Valve remains the lowest-AP class after improvement, indicating that rare compact control components are still affected by background confusion and limited training examples.

**Figure 7:**
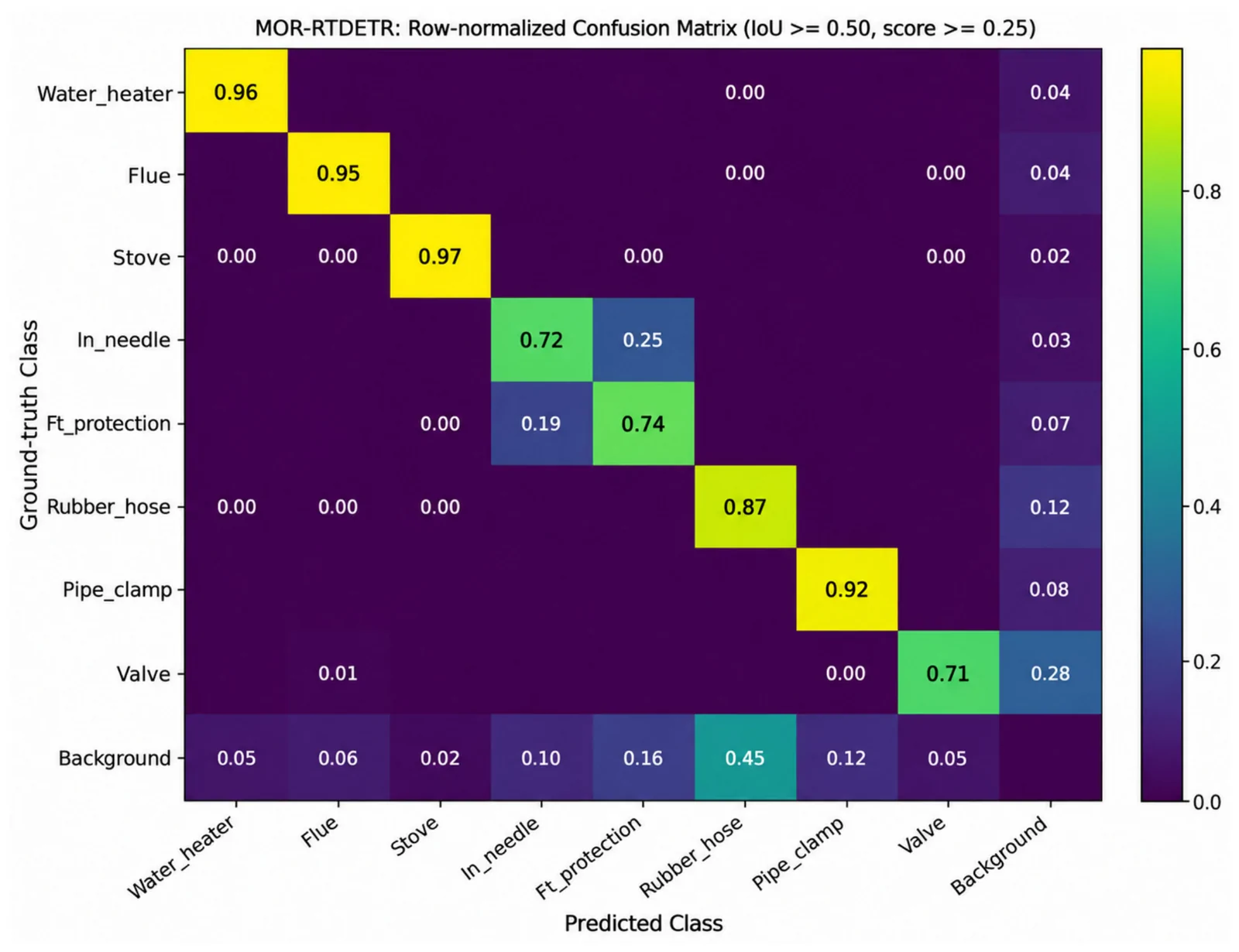
Row-normalized confusion matrix of MOR-RTDETR. Rows denote ground-truth categories, and columns denote predicted categories.

Although Valve accounts for only 2.88% of the annotated instances, no class-specific resampling or loss reweighting strategy was introduced in this study. The primary objective was to evaluate the contributions of GGME, BLGIE, and SAPR under a consistent training protocol, while retaining the original classification loss and dataset distribution. Introducing class-specific balancing strategies would constitute an additional training intervention and could complicate the attribution of performance improvements to the proposed modules. Nevertheless, Valve remains the lowest-performing category despite its AP improvement from 0.4140 to 0.4662. This suggests that class imbalance, together with small object size and background ambiguity, may continue to limit detection performance. Investigating class-balanced sampling or loss reweighting for rare components is therefore a worthwhile direction for future work. To further analyze class-wise recognition performance and prediction confusion, the row-normalized confusion matrix of MOR-RTDETR is presented in Figure 7.

The confusion matrix of MOR-RTDETR shows that most predictions are concentrated along the diagonal, particularly for Water_heater, Flue, Stove, Rubber_hose, and Pipe_clamp. Residual ambiguity remains mainly between In_needle and Ft_protection, while Valve still shows sensitivity to background confusion. These patterns indicate that MOR-RTDETR improves overall recognition and localization but still faces difficulty in compact, visually similar, and context-dependent components.

### 3.5. Ablation and Hyperparameter Sensitivity Analysis

#### 3.5.1. Component and Regression-Loss Ablation

Table 8 provides a controlled evaluation of the individual and combined contributions of GGME, BLGIE, and SAPR under identical training settings. Compared with RT-DETR-R18, GGME alone improves mAP from 0.6767 to 0.6954 (+1.87 percentage points) and High-AR AP from 0.5480 to 0.5861 (+3.81 points), supporting its contribution to morphology-aware feature representation. BLGIE alone increases occlusion-tag AP from 0.6280 to 0.6583 (+3.03 points), suggesting improved feature representation in images containing partial visual evidence. SAPR alone improves AP75 from 0.7449 to 0.7639 (+1.90 points) and High-AR AP from 0.5480 to 0.5742 (+2.62 points), demonstrating the benefit of short-axis-aware regression supervision. Furthermore, adding SAPR to the GGME + BLGIE configuration increases mAP from 0.7036 to 0.7108 (+0.72 points), AP75 from 0.7814 to 0.7987 (+1.73 points), and APsmall from 0.4558 to 0.4928 (+3.70 points). These results provide quantitative evidence of the complementary contributions of the proposed modules within the RT-DETR-R18 framework. The occlusion-tagged subset contains non-exclusive image-level labels and is therefore used for diagnostic evaluation rather than instance-level occlusion assessment. Since SAPR modifies only the training objective, minor FPS differences in the SAPR-only experiment are attributable to measurement variability rather than additional inference operations.

To further investigate the rationale for retaining L1 and GIoU as the reference regression objectives and introducing SAPR as an additional supervision term, we conducted a controlled comparison with two alternative IoU-based losses, Focal-EIoU [40] and Wise-IoU v3 [41]. L1 provides coordinate-wise localization supervision, while GIoU incorporates two-dimensional bounding-box overlap and an enclosing-region penalty. However, neither objective explicitly emphasizes localization sensitivity along the short axis of elongated targets. We therefore retain the original RT-DETR regression formulation to isolate the additional contribution of SAPR and compare it against alternative regression configurations. All four configurations use the same GGME–BLGIE feature enhancement architecture, with the regression objective as the experimental variable. Focal-EIoU is evaluated with γ = 0.5, while Wise-IoU v3 uses α = 1.9 and δ = 3.0. The comparison results are presented in Table 9.

As shown in Table 9, both Focal-EIoU and Wise-IoU v3 improve detection performance over the original L1 + GIoU configuration, indicating that alternative IoU-based regression formulations are beneficial for the evaluated task. Nevertheless, the proposed L1 + GIoU + SAPR configuration achieves the highest mean performance across all four evaluation metrics, with an mAP of 0.7108, AP75 of 0.7987, APsmall of 0.4928, and High-AR AP of 0.6406. Compared with Wise-IoU v3, which achieves the highest mAP among the alternative loss configurations, the proposed approach improves mAP by 0.32 percentage points, AP75 by 1.13 percentage points, and High-AR AP by 2.77 percentage points. Compared with Focal-EIoU, the improvements in AP75 and High-AR AP are 0.98 and 2.38 percentage points, respectively. These results suggest that the additional short-axis-aware projection supervision is beneficial for accurate localization, particularly for elongated objects. Importantly, this comparison supports the effectiveness of the combined L1 + GIoU + SAPR objective within the evaluated household gas facility detection task, rather than establishing the universal superiority of GIoU over alternative IoU-based losses.

#### 3.5.2. Internal Sub-Component Ablation

To further investigate the contributions of the internal mechanisms within GGME, BLGIE, and SAPR, six sub-component ablation configurations were evaluated based on the full MOR-RTDETR model. Each configuration selectively disables one internal mechanism while retaining the remaining components and the same training and evaluation protocol. Specifically, the experiments examine the directional sampling branches and gradient-guided branch selection in GGME, bidirectional cross-calibration and discrepancy-guided reliability fusion in BLGIE, and the projected regression and shape-consistency terms in SAPR. The corresponding experimental results are summarized in Table 10.

As shown in Table 10, removing the internal sub-components generally reduces detection performance compared with the complete MOR-RTDETR model. For GGME, removing the directional sampling branches decreases High-AR AP from 0.6406 to 0.5964 and mAP from 0.7108 to 0.7019. Removing gradient guidance reduces High-AR AP to 0.6218, indicating that both directional feature sampling and gradient-guided branch selection contribute to morphology-aware representation.

For BLGIE, removing bidirectional calibration reduces Occlusion-tag AP from 0.6819 to 0.6589, whereas removing discrepancy-guided fusion decreases it to 0.6634. These results suggest that both mechanisms contribute to feature representation under challenging visual conditions.

For SAPR, removing the projected regression term decreases AP75 from 0.7987 to 0.7862 and High-AR AP from 0.6406 to 0.6145. Removing the shape-consistency term also leads to performance reductions, although their magnitudes are smaller. Since SAPR operates only during training, these modifications do not affect inference-time model complexity.

Overall, the results provide additional evidence that the internal mechanisms make complementary contributions to the detection performance of MOR-RTDETR under the evaluated experimental settings.

#### 3.5.3. Hyperparameter Sensitivity Analysis

To investigate the influence of key hyperparameters on the detection performance of MOR-RTDETR, a one-factor-at-a-time sensitivity analysis was conducted. Five representative hyperparameters were examined: the channel-reduction ratio (*ρ*) and DSConv kernel length (*k*) in GGME, the MLP reduction ratio (*r*) in BLGIE, and the shape-consistency weight (*λ_s_*) and short-axis sensitivity coefficient (*γ*) in SAPR. In each experiment, only one hyperparameter was varied, while the remaining model configurations, training settings, and evaluation protocol were kept unchanged. Each configuration was evaluated using three independent runs with random seeds 42, 123, and 2024, consistent with the experimental protocol in Section 2.7. The results are summarized in Table 11.

The GGME sensitivity results indicate that the channel-reduction ratio affects both detection performance and computational efficiency. Reducing *ρ* from 0.50 to 0.25 decreases the computational cost from 63.7 to 60.2 GFLOPs but also reduces mAP from 0.7108 to 0.7033. Increasing *ρ* to 0.75 achieves comparable overall accuracy but increases model complexity to 68.1 GFLOPs and reduces throughput to 67.8 FPS. Regarding the DSConv kernel length, *k* = 9 achieves the highest mean mAP and High-AR AP among the tested values, suggesting a favorable balance between morphology-adaptive feature sampling and computational overhead.

For BLGIE, an MLP reduction ratio of *r* = 4 achieves the highest mean mAP among the tested settings, while *r* = 2 yields a slightly higher Occlusion-tag AP (0.6825 versus 0.6819). Given the small difference relative to run-to-run variability, the default ratio is retained for its favorable overall performance. For SAPR, the default shape-consistency weight *λ_s_* = 0.50 provides the highest mean mAP and AP_75_ among the tested weights, although *λ_s_* = 0.75 produces a marginally higher High-AR AP. Similarly, the short-axis sensitivity coefficient *γ* = 1.5 achieves higher mean mAP, AP_75_, and High-AR AP than *γ* = 1.0 or 2.0 within the evaluated parameter range.

Overall, the sensitivity analysis suggests that the adopted hyperparameter configuration provides a favorable balance between detection accuracy and computational efficiency within the evaluated parameter ranges. The results also indicate that MOR-RTDETR exhibits some sensitivity to the selected architectural and regression-related hyperparameters, particularly in high-aspect-ratio localization and strict bounding-box accuracy. Nevertheless, several performance differences are small relative to the observed standard deviations, and the present experiments do not establish global hyperparameter optimality.

### 3.6. Data-Supported Qualitative Results and Failure Analysis

**Figure 8:**
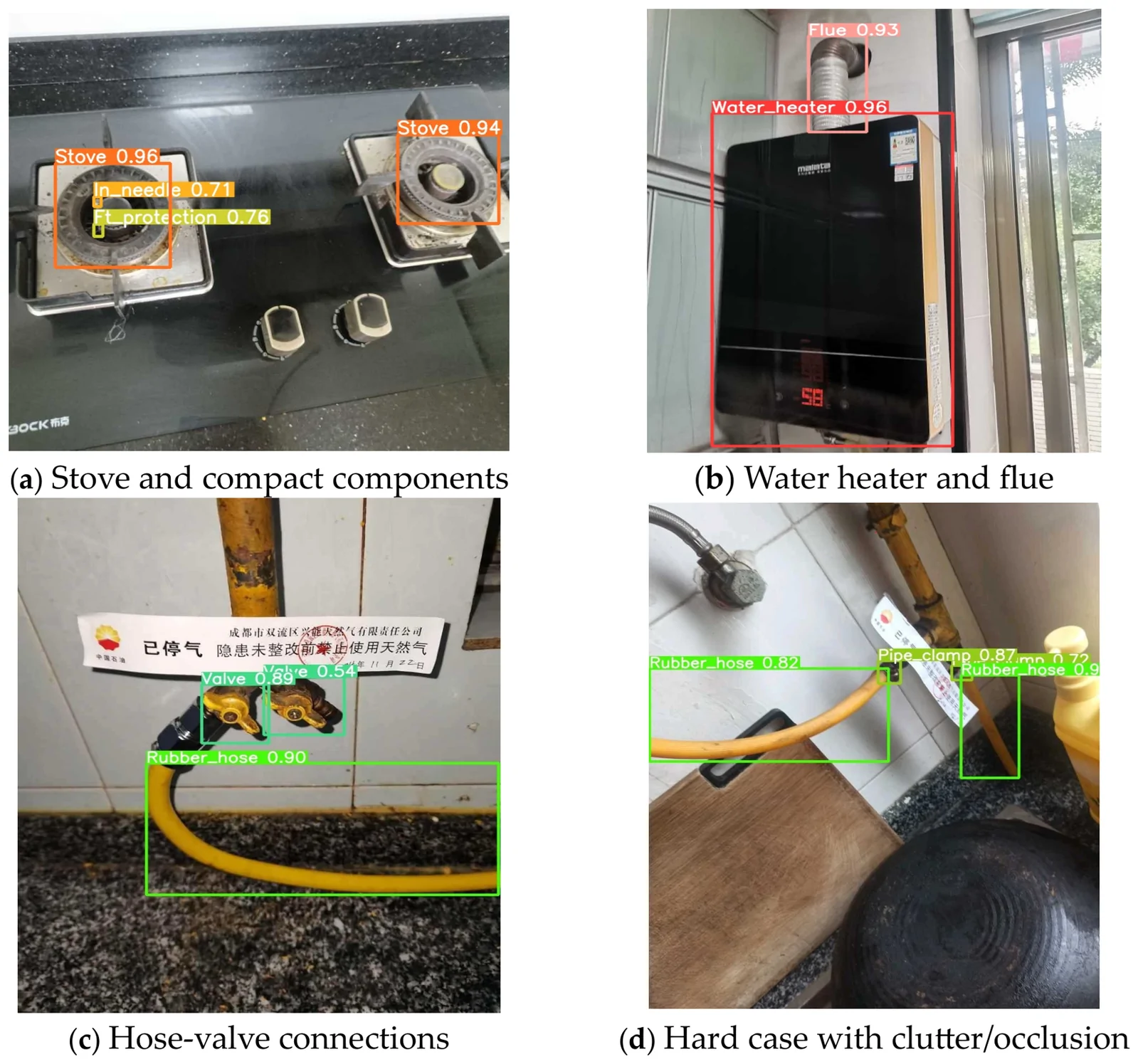
Representative qualitative detections produced by MOR-RTDETR. The examples cover stove components, water-heater/flue scenes, hose-valve-pipe connections, and hard cases with clutter or partial occlusion. The figure is used as an illustrative visualization of the quantitative trends summarized in Table 12, and privacy-sensitive header information in the source images has been cropped out for manuscript presentation. The Chinese text visible in panel (**c**) is part of the original on-site gas-equipment labeling and is unrelated to the detection results or their scientific interpretation.

Representative examples are retained in Figure 8 to illustrate typical detection scenes, but the qualitative interpretation is supported primarily by the quantitative indicators summarized in Table 12. The table links each visual behavior to a concise metric-based observation rather than relying on selected images alone.

Table 12 provides the quantitative basis for interpreting Figure 8. Improvements in AP75, APsmall, High-AR AP, and occlusion-tag AP correspond to stricter boundary alignment, stronger compact-object responses, better high-aspect-ratio localization, and improved performance on image-level occlusion-tagged scenes.

Consistent with the data-supported analysis in Table 12, the qualitative examples are interpreted conservatively. The remaining failure cases include near-complete occlusion, severe motion blur, extreme metallic overexposure, background lines resembling hoses, highly unusual layouts, and extremely small or truncated targets. These limitations correspond to missing visual evidence, horizontal-box ambiguity, contextual bias, class imbalance, or insufficient spatial resolution.

More specifically, the residual confusion between In_needle and Ft_protection observed in Figure 7 may be related to their small apparent sizes, spatial proximity, and similar local visual structures, which make reliable category discrimination challenging under limited image resolution. For Valve, the relatively small number of annotated instances (2.88% of the dataset, as shown in Table 1), together with cluttered backgrounds and visually similar surrounding pipe fittings, may contribute to missed detections and background-related false positives. These possible failure mechanisms are consistent with the class-wise performance and confusion-matrix observations, although their individual contributions have not been independently quantified.

## 4. Discussion

### 4.1. Overall Performance and Mechanism Analysis

The overall results indicate that MOR-RTDETR should be interpreted as a task-oriented improvement of RT-DETR-R18 for household gas facility localization, rather than as a universally optimal detector. The mAP gain of 3.41 percentage points is accompanied by a 5.38-point AP75 gain and an 8.68-point APsmall gain, showing that the improvement is concentrated in stricter localization and compact-object detection. The representative-detector comparison in Table 6 further situates MOR-RTDETR within this dataset-specific protocol: the model obtains stronger accuracy than the compared YOLO, two-stage, and Transformer-based alternatives on this validation set while maintaining real-time throughput. The data-supported qualitative analysis in Table 12 links these numerical gains to typical visual phenomena in Figure 8, including better boundary alignment, stronger small-component responses, improved high-aspect-ratio localization, and more reliable predictions on image-level occlusion-tagged scenes.

The class-wise comparison clarifies the distribution of gains. Water_heater, Stove, and Flue already obtain high baseline AP, so their improvements are relatively small. In contrast, Pipe_clamp, Ft_protection, Valve, and Rubber_hose exhibit larger improvements because they are compact, context-dependent, elongated, or weakly separated from adjacent structures. Although Rubber_hose is the most direct motivation for morphology-aware and short-axis-aware design, Pipe_clamp also benefits substantially because its small size, weak boundary, and surrounding pipe context make it sensitive to local contour preservation, reliability-guided feature fusion, and stricter boundary localization. This pattern is consistent with the design goals of GGME, BLGIE, and SAPR and indicates that the proposed modules are not limited to a single elongated category.

The MOR-RTDETR confusion matrix reveals the main residual difficulty of compact and context-dependent components. In_needle and Ft_protection still show mutual confusion, and Valve remains sensitive to background responses. The occlusion-related conclusion is therefore tied specifically to the occlusion-tagged validation subset containing 1255 image-level, non-exclusive tagged images. Because these images may simultaneously contain other degradations, the observed gain should be interpreted as improved detection on images containing partial visual evidence rather than isolated causal evidence of occlusion modeling. BLGIE should therefore be interpreted as reliability-guided local–global feature fusion, not as an instance-level occlusion-understanding or occlusion-localization module.

The AP75 and High-AR AP improvements indicate that SAPR strengthens regression supervision for high-aspect-ratio horizontal boxes. SAPR decomposes box alignment into one-dimensional projected overlap terms and emphasizes the ground-truth short-axis direction during training. It should not be interpreted as recovering the physical contour, centerline, or curved geometry of rubber hoses; those representations would require rotated boxes, instance masks, keypoints, or centerline annotations.

### 4.2. Accuracy-Efficiency Trade-Off and Application Scope

MOR-RTDETR increases the model size from 19.9 M to 22.3 M parameters and the computational cost from 57.8 G to 63.7 G FLOPs. The measured throughput decreases from 76.2 to 72.3 FPS under the batch-1 FP32 protocol. This overhead is moderate relative to the gains in APsmall and AP75, but the model should still be selected according to the latency and memory constraints of the target inspection terminal. The method is intended to support image recording and visible component localization during household gas inspection. It cannot independently determine gas leakage, sealing integrity, component aging, or installation compliance. These engineering limitations define the application scope of MOR-RTDETR as an inspection-assistance detector rather than a complete gas-safety diagnosis system.

### 4.3. Limitations and Future Work

Several limitations remain. First, Rubber_hose is the main elongated category in the current dataset, and the generalization of GGME and SAPR to other deformable or tubular objects requires further validation. Second, the available degradation labels are non-exclusive image-level tags; therefore, the corresponding results cannot precisely measure instance-level occlusion severity or the causal effect of an individual degradation factor. Third, horizontal bounding boxes provide limited geometric descriptions for strongly curved or obliquely oriented hoses. SAPR improves box-regression supervision but does not recover object contours or centerlines. Future work will investigate rotated boxes, instance segmentation, keypoint or centerline representations, and object-level degradation annotations.

In addition, the current dataset was acquired from a limited range of household environments and imaging devices. Therefore, the reported performance should be interpreted within the evaluated residential inspection scenarios, and generalization to other geographical regions, imaging devices, or operational settings has not yet been established. Cross-region, cross-device, and prospective field evaluations are needed before broader deployment [45]. Model pruning, quantization, multimodal sensing, and hardware-aware optimization will also be explored for resource-constrained inspection terminals.

Another limitation concerns the evaluation protocol. The same validation set was used for checkpoint selection and performance reporting, which may introduce optimistic bias into the reported results. Although the household-scene-level split reduces overlap between training and validation samples, it does not eliminate potential model-selection bias. In the absence of an independent test set, the present results should be interpreted as comparative evidence within the evaluated residential inspection dataset rather than unbiased estimates of generalization performance. Future work will incorporate an independent scene-level or site-level test set to provide a more rigorous assessment of the proposed method.

## 5. Conclusions

This study proposes MOR-RTDETR, a task-oriented adaptation of RT-DETR-R18 for household gas facility visual sensing. The model integrates three complementary improvements into the baseline end-to-end detection pipeline. GGME combines standard and directional dynamic-sampling branches with fixed feature-gradient guidance to enhance elongated-structure representation. BLGIE performs reliability-guided bidirectional calibration between local structural evidence and global semantic information, and its occlusion-related analysis is limited to image-level occlusion-tagged validation images. SAPR introduces short-axis-weighted projected GIoU and elongation-gated shape consistency to strengthen regression supervision for high-aspect-ratio horizontal boxes while retaining the original L1 and GIoU objectives.

Under the fixed scene-level split and unified evaluation protocol, MOR-RTDETR improves the RT-DETR-R18 baseline from 0.6767 to 0.7108 COCO-style mAP, from 0.8543 to 0.8782 AP50, from 0.7449 to 0.7987 AP75, and from 0.4060 to 0.4928 APsmall. The representative detector comparison further indicates that the proposed model provides a favorable accuracy-efficiency trade-off on this household gas facility validation set. The data-supported qualitative analysis shows that these gains correspond to stricter boundary alignment, better compact-object detection, improved high-aspect-ratio localization, and stronger performance on image-level occlusion-tagged scenes. The largest class-wise gains occur for Pipe_clamp, Ft_protection, Valve, and Rubber_hose, which are affected by compact scale, contextual ambiguity, elongated morphology, and weak boundaries. Overall, these results support MOR-RTDETR as a task-oriented adaptation of RT-DETR-R18 for assisting household gas facility localization within the evaluated residential inspection scenarios, while broader deployment requires further external validation.

## Figures and Tables

**Table 1:** Category-level instance distribution and task characteristics of the 8-class household gas facility dataset.

Category	Training Instances	Validation Instances	Total Instances	Total Proportion	Main Task Characteristic
Water_heater	6279	1607	7886	16.72%	Large regular appliance; high visual salience
Flue	4914	1231	6145	13.03%	Elongated exhaust-related component; viewpoint variation
Stove	4802	1205	6007	12.74%	Large regular appliance; cluttered surroundings
In_needle	3511	879	4390	9.31%	Small and fine-structured inlet component
Ft_protection	2844	676	3520	7.46%	Small protection component; visually similar to adjacent structures
Rubber_hose	9450	2350	11,800	25.02%	Flexible, slender, curved, and high-aspect-ratio component
Pipe_clamp	4832	1232	6064	12.86%	Compact fixation component; weak boundary and contextual ambiguity
Valve	1056	301	1357	2.88%	Rare compact control component; high background confusion risk
All categories	37,688	9481	47,169	100.00%	Eight-class household gas facility detection

Note: The instance counts are obtained from the fixed scene-level split used in this study. The task-characteristic descriptions are used to motivate the MOR-RTDETR design and are not additional manual object attributes.

**Table 2:** Validation subsets with image-level degradation tags.

Degradation Tag	Definition	Validation Images	Proportion
Blur	Defocus or motion blur	1085	24.87%
Reflection/overexposure	Metallic reflection or overexposed region	1031	23.63%
Stain/dust	Visible stain, dust, or contamination	818	18.75%
Occlusion	Image contains visible occlusion or partial blocking	1255	28.77%
Low illumination/shadow	Low light or strong shadow	861	19.73%
Background clutter	Cluttered background or dense surrounding structures	1022	23.42%

Note: Percentages are calculated with 4363 validation images as the denominator. The degradation tags are non-exclusive image-level labels and are not instance-level occlusion annotations; therefore, percentages do not sum to 100%.

**Table 3:** Experimental environment and training configuration for the controlled RT-DETR-R18 and MOR-RTDETR experiments.

Setting	Value
Optimizer	stochastic gradient descent (SGD; Nesterov momentum = 0.937)
Weight decay	5 × 10^−4^
Initial learning rate	0.01
Final learning rate	1 × 10^−4^
learning rate (LR) schedule	Linear warm-up (1000 iters) + linear decay
Batch size	16
Data-loading workers	4
Epochs	300
Early-stopping patience	15 epochs
Pretrained weights	RT-DETR-R18vd pretrained on Microsoft Common Objects in Context (COCO; official release v1.0)
Random seeds	42, 123, 2024 (3 independent runs)
Input resolution	640 × 640
Augmentation	HFlip (p = 0.5), scale [0.5, 1.5], translate [−0.1, 0.1], color jitter (brightness/contrast/saturation [0.8, 1.2], hue [−0.1, 0.1]), affine (rotation [−5°, 5°], shear [−0.05, 0.05])
Hardware	NVIDIA GeForce RTX 4060 Laptop graphics processing unit (GPU; NVIDIA Corporation, Santa Clara, CA, USA), 8 GB
Software	Python 3.10.13, PyTorch 2.1.2, CUDA 12.1, CUDA Deep Neural Network library (cuDNN) 8.9.2
FPS protocol	Batch 1, 32-bit floating point (FP32), 50 warm-up + 200 timed iterations, including preprocessing and post-processing

**Table 4:** Overall performance and complexity comparison on the fixed validation set.

Method	mAP	AP50	AP75	APsmall	Params (M)	FLOPs (G)	FPS
RT-DETR-R18	0.6767 ± 0.0029	0.8543 ± 0.0025	0.7449 ± 0.0031	0.4060 ± 0.0047	19.9	57.8	76.2
MOR-RTDETR	0.7108 ± 0.0026	0.8782 ± 0.0021	0.7987 ± 0.0027	0.4928 ± 0.0043	22.3	63.7	72.3

Note: Accuracy metrics are reported as mean ± standard deviation (SD) over three random seeds. Parameters (Params) and FLOPs are measured once at 640 × 640 input resolution. FPS is measured with batch size 1 in FP32 using 50 warm-up iterations and 200 timed iterations, including preprocessing and post-processing.

**Table 5:** Implementation details and training configurations of the evaluated detectors.

Detector	Codebase	Config. Strategy	Optimizer	LR	WD	Batch (GPU/Eff.)	Max Epochs
Faster R-CNN-R50 [7]	MMDetection	Reference 2×, LR-adapted	SGD	0.01	1 × 10^−4^	8/8	24
YOLOv8n [43]	Ultralytics	Standard, SGD-specified	SGD	0.01	5 × 10^−4^	16/16	100
YOLOv8s [43]	Ultralytics	Standard, SGD-specified	SGD	0.01	5 × 10^−4^	16/16	100
YOLOv10n [44]	THU-MIG YOLOv10	Standard, SGD-specified	SGD	0.01	5 × 10^−4^	16/16	100
YOLOv10s [44]	THU-MIG YOLOv10	Extended training with early stopping	SGD	0.01	5 × 10^−4^	16/16	300
Deformable DETR-R50 [21]	MMDetection	Reference-based, accumulation	AdamW	2 × 10^−4^	1 × 10^−4^	4/16	50
DINO-R50 [22]	IDEA-Research	24-epoch, accumulation	AdamW	1 × 10^−4^	1 × 10^−4^	4/16	24
RT-DETR-R18 [23]	RT-DETR/PyTorch	Controlled, modified	SGD	0.01	5 × 10^−4^	16/16	300
MOR-RTDETR (Ours)	Modified RT-DETR	Same as baseline	SGD	0.01	5 × 10^−4^	16/16	300

Note: LR denotes the initial learning rate, and WD denotes weight decay. Batch sizes are reported as per-GPU/effective batch sizes. Gradient accumulation was applied to Deformable DETR-R50 and DINO-R50 with an accumulation factor of four. Max Epochs denotes the maximum permitted training epochs rather than the actual number of epochs completed. When early stopping is enabled, training may terminate before reaching this limit. RT-DETR-R18, YOLOv10s, and MOR-RTDETR were evaluated with a common maximum training budget of 300 epochs and early stopping with a patience of 15 epochs.

**Table 6:** Comparison with mainstream object detectors under the same data split and evaluation protocol.

Method	mAP	AP50	AP75	APsmall	Params (M)	FLOPs (G)	FPS
Faster R-CNN-R50	0.628 ± 0.004	0.804 ± 0.004	0.691 ± 0.005	0.383 ± 0.006	41.5	134.2	18.6
YOLOv8n	0.643 ± 0.004	0.818 ± 0.003	0.708 ± 0.004	0.385 ± 0.006	3.2	8.7	132.4
YOLOv8s [43]	0.664 ± 0.004	0.836 ± 0.003	0.732 ± 0.004	0.413 ± 0.005	11.2	28.6	104.8
YOLOv10n [44]	0.656 ± 0.004	0.829 ± 0.004	0.719 ± 0.004	0.402 ± 0.006	2.3	6.7	141.6
YOLOv10s [44]	0.6863 ± 0.0021	0.8564 ± 0.0027	0.7578 ± 0.0032	0.4405 ± 0.0045	7.2	21.6	112.7
Deformable DETR-R50	0.650 ± 0.005	0.822 ± 0.004	0.716 ± 0.005	0.396 ± 0.007	40.2	173.0	15.2
DINO-R50	0.682 ± 0.004	0.857 ± 0.004	0.750 ± 0.005	0.433 ± 0.006	47.0	215.4	11.8
RT-DETR-R18	0.677 ± 0.003	0.854 ± 0.003	0.745 ± 0.003	0.406 ± 0.005	19.9	57.8	76.2
MOR-RTDETR	0.711 ± 0.003	0.878 ± 0.002	0.799 ± 0.003	0.493 ± 0.004	22.3	63.7	72.3

Note: Accuracy metrics are reported as mean ± standard deviation (SD) over three seeds. Params are reported in M, FLOPs in G, and FPS under the same batch-1 FP32 protocol as Table 4. YOLOv8 is cited through the Ultralytics documentation because no formal YOLOv8 paper has been released. The YOLOv10s results correspond to the additional experiments conducted with a maximum training budget of 300 epochs and an early-stopping patience of 15 epochs, matching the maximum epoch budget and early-stopping criterion of RT-DETR-R18 and MOR-RTDETR. The remaining comparison detectors retain their respective architecture-specific training configurations, as detailed in Table 5.

**Table 7:** Class-wise COCO-style AP comparison between RT-DETR-R18 and MOR-RTDETR, with AP50 values for MOR-RTDETR.

Category	RT-DETR-R18 AP	MOR-RTDETR AP	ΔAP	MOR-RTDETR AP50
Water_heater	0.9115	0.9168	+0.0053	0.9704
Flue	0.8210	0.8314	+0.0104	0.9547
Stove	0.9209	0.9237	+0.0028	0.9809
In_needle	0.6934	0.7196	+0.0262	0.9316
Ft_protection	0.5067	0.5592	+0.0525	0.7952
Rubber_hose	0.5693	0.6097	+0.0404	0.8045
Pipe_clamp	0.5766	0.6598	+0.0832	0.9103
Valve	0.4140	0.4662	+0.0522	0.6783
All classes	0.6767	0.7108	+0.0341	0.8782

Note: Class-wise AP values are averaged over three independent runs with random seeds 42, 123, and 2024. ΔAP represents the difference between the corresponding mean AP values of MOR-RTDETR and RT-DETR-R18.

**Table 8:** Main component ablation under identical training settings.

Variant	GGME	BLGIE	SAPR	mAP	AP75	APsmall	High-AR AP	Occlusion-Tag AP	Params (M)	FLOPs (G)	FPS
Baseline RT-DETR	–	–	–	0.6767 ± 0.0029	0.7449 ± 0.0031	0.4060 ± 0.0047	0.5480 ± 0.0051	0.6280 ± 0.0048	19.9	57.8	76.2
GGME only	✓	–	–	0.6954 ± 0.0031	0.7668 ± 0.0034	0.4279 ± 0.0050	0.5861 ± 0.0047	0.6396 ± 0.0049	21.4	62.3	73.7
BLGIE only	–	✓	–	0.6909 ± 0.0033	0.7597 ± 0.0036	0.4214 ± 0.0048	0.5627 ± 0.0053	0.6583 ± 0.0045	20.8	59.2	74.6
SAPR only	–	–	✓	0.6869 ± 0.0030	0.7639 ± 0.0032	0.4187 ± 0.0046	0.5742 ± 0.0048	0.6327 ± 0.0050	19.9	57.8	75.9
GGME + BLGIE	✓	✓	–	0.7036 ± 0.0028	0.7814 ± 0.0030	0.4558 ± 0.0045	0.6036 ± 0.0042	0.6664 ± 0.0041	22.3	63.7	72.6
GGME + SAPR	✓	–	✓	0.7061 ± 0.0027	0.7886 ± 0.0029	0.4709 ± 0.0044	0.6208 ± 0.0041	0.6463 ± 0.0047	21.4	62.3	73.4
BLGIE + SAPR	–	✓	✓	0.6998 ± 0.0030	0.7821 ± 0.0031	0.4493 ± 0.0047	0.5876 ± 0.0046	0.6711 ± 0.0040	20.8	59.2	74.1
MOR-RTDETR	✓	✓	✓	0.7108 ± 0.0026	0.7987 ± 0.0027	0.4928 ± 0.0043	0.6406 ± 0.0046	0.6819 ± 0.0042	22.3	63.7	72.3

Note: “✓” indicates that the corresponding module is included in the model, while “–” indicates that the module is not included.

**Table 9:** Comparison of different bounding-box regression loss configurations under the same GGME–BLGIE architecture.

Group	Regression Loss Configuration	mAP	AP75	APsmall	High-AR AP
A	L1 + GIoU [38]	0.7036 ± 0.0028	0.7814 ± 0.0030	0.4558 ± 0.0045	0.6036 ± 0.0042
B	L1 + Focal-EIoU [40] (γ = 0.5)	0.7068 ± 0.0029	0.7889 ± 0.0031	0.4682 ± 0.0047	0.6168 ± 0.0045
C	L1 + Wise-IoU v3 [41] (α = 1.9, δ = 3.0)	0.7076 ± 0.0028	0.7874 ± 0.0031	0.4785 ± 0.0046	0.6129 ± 0.0047
D	L1 + GIoU + SAPR	0.7108 ± 0.0026	0.7987 ± 0.0027	0.4928 ± 0.0043	0.6406 ± 0.0046

Note: All configurations employ the same GGME–BLGIE architecture and are evaluated under the same training and evaluation protocol, with only the regression-loss configuration varied. Results are reported as mean ± standard deviation (SD) over three independent runs with random seeds 42, 123, and 2024. Group A corresponds to the GGME + BLGIE configuration without SAPR in Table 8, whereas Group D corresponds to the complete MOR-RTDETR model. The classification loss and Hungarian matching cost remain unchanged across the four configurations.

**Table 10:** Internal sub-component ablation of GGME, BLGIE, and SAPR.

Module	Variant	mAP	AP_75_	High-AR AP	Occ.-Tag AP	Params (M)	FLOPs (G)
All	Full MOR-RTDETR	0.7108 ± 0.0026	0.7987 ± 0.0027	0.6406 ± 0.0046	0.6819 ± 0.0042	22.3	63.7
GGME	w/o directional branches	0.7019 ± 0.0030	0.7856 ± 0.0032	0.5964 ± 0.0048	0.6738 ± 0.0043	21.2	60.4
GGME	w/o gradient guidance	0.7069 ± 0.0028	0.7934 ± 0.0030	0.6218 ± 0.0047	0.6785 ± 0.0044	22.3	63.6
BLGIE	w/o bidirectional calibration	0.7076 ± 0.0029	0.7921 ± 0.0031	0.6342 ± 0.0045	0.6589 ± 0.0046	22.1	63.5
BLGIE	w/o discrepancy-guided fusion	0.7083 ± 0.0027	0.7940 ± 0.0029	0.6358 ± 0.0046	0.6634 ± 0.0044	22.1	63.4
SAPR	w/o projection term (ℒ_proj_)	0.7054 ± 0.0029	0.7862 ± 0.0031	0.6145 ± 0.0048	0.6712 ± 0.0043	22.3	63.7
SAPR	w/o shape-consistency term (ℒ_shape_)	0.7079 ± 0.0027	0.7943 ± 0.0029	0.6274 ± 0.0046	0.6789 ± 0.0042	22.3	63.7

Note: Accuracy results are reported as mean ± standard deviation (SD) over three independent runs using random seeds 42, 123, and 2024. Each ablation configuration removes or replaces one internal mechanism while retaining the remaining components under the same training and evaluation protocol. Params and FLOPs refer to inference-time model complexity measured at an input resolution of 640 × 640. SAPR modifies only the training loss and therefore does not introduce additional inference-time parameters or operations. The occlusion-tagged subset is defined using non-exclusive image-level labels rather than instance-level occlusion annotations.

**Table 11:** Sensitivity analysis of key hyperparameters in MOR-RTDETR.

Varied Parameter	Value	mAP	AP_75_	AP_small_	High-AR AP	Occ.-Tag AP	Params (M)	FLOPs (G)	FPS
Default	—	0.7108 ± 0.0026	0.7987 ± 0.0027	0.4928 ± 0.0043	0.6406 ± 0.0046	0.6819 ± 0.0042	22.3	63.7	72.3
*ρ* (GGME)	0.25	0.7033 ± 0.0031	0.7890 ± 0.0034	0.4764 ± 0.0048	0.6143 ± 0.0050	0.6757 ± 0.0045	21.1	60.2	74.6
*ρ* (GGME)	0.75	0.7105 ± 0.0028	0.7984 ± 0.0030	0.4935 ± 0.0045	0.6396 ± 0.0044	0.6813 ± 0.0041	23.9	68.1	67.8
*k* (GGME)	7	0.7067 ± 0.0029	0.7938 ± 0.0032	0.4883 ± 0.0044	0.6246 ± 0.0049	0.6796 ± 0.0044	22.1	63.0	73.1
*k* (GGME)	11	0.7076 ± 0.0030	0.7931 ± 0.0033	0.4870 ± 0.0047	0.6282 ± 0.0052	0.6787 ± 0.0046	22.5	64.5	70.9
*r* (BLGIE)	2	0.7093 ± 0.0025	0.7967 ± 0.0028	0.4903 ± 0.0042	0.6381 ± 0.0047	0.6825 ± 0.0043	22.4	63.7	72.0
*r* (BLGIE)	8	0.7078 ± 0.0029	0.7942 ± 0.0031	0.4845 ± 0.0046	0.6385 ± 0.0045	0.6723 ± 0.0045	22.2	63.7	72.5
*λ_s_* (SAPR)	0.25	0.7088 ± 0.0027	0.7959 ± 0.0028	0.4909 ± 0.0044	0.6318 ± 0.0046	0.6805 ± 0.0041	22.3	63.7	72.4
*λ_s_* (SAPR)	0.75	0.7081 ± 0.0029	0.7939 ± 0.0032	0.4891 ± 0.0045	0.6412 ± 0.0049	0.6797 ± 0.0044	22.3	63.7	72.1
*γ* (SAPR)	1.0	0.7074 ± 0.0026	0.7916 ± 0.0029	0.4888 ± 0.0043	0.6269 ± 0.0048	0.6803 ± 0.0043	22.3	63.7	72.5
*γ* (SAPR)	2.0	0.7063 ± 0.0030	0.7895 ± 0.0033	0.4873 ± 0.0046	0.6250 ± 0.0050	0.6793 ± 0.0042	22.3	63.7	72.2

Note: Accuracy results are reported as mean ± standard deviation (SD) over three independent runs. Only one hyperparameter was varied at a time, while the other settings remained fixed at their default values (ρ = 0.50, k = 9, r = 4, λ_s = 0.50, and γ = 1.5). Params and FLOPs were calculated at an input resolution of 640 × 640. FPS is reported under the unified batch-1 FP32 inference protocol described in Section 3.1. High-AR AP denotes the average precision of high-aspect-ratio objects, while Occlusion-tag AP is evaluated on the non-exclusive image-level occlusion-tagged subset.

**Table 12:** Quantitative evidence corresponding to qualitative detection behaviors.

Qualitative Aspect	Metric	RT-DETR-R18 → > MOR-RTDETR	Interpretation
Overall validation trend	mAP	0.6767 ± 0.0029 → 0.7108 ± 0.0026 (+3.41 points)	Improved overall detection reliability
Strict localization	AP75	0.7449 ± 0.0031 → 0.7987 ± 0.0027 (+5.38 points)	Better boundary alignment under stricter IoU
Small-object response	APsmall	0.4060 ± 0.0047 → 0.4928 ± 0.0043 (+8.68 points)	Stronger compact-object detection
Elongated-object localization	High-AR AP	0.5480 → 0.6406 (+9.26 points)	Improved high-aspect-ratio localization
Occlusion-tagged scenes	Occlusion-tag AP	0.6280 → 0.6819 (+5.39 points)	More reliable detection on image-level occlusion-tagged images
Weak-boundary fixation component	Pipe_clamp AP	0.5766 → 0.6598 (+8.32 points)	Largest class-wise gain for compact fixation structures
Residual rare-class difficulty	Valve AP	0.4140 → 0.4662 (+5.22 points)	Persistent difficulty from rarity and background confusion

Note: The arrow (→) indicates the performance change from the baseline RT-DETR-R18 to the proposed MOR-RTDETR model.

## Data Availability

The data presented in this study are available from the corresponding author upon reasonable request. The raw dataset is not publicly available due to the data management arrangements established for this study.
